# Biomechanical Performance Factors in the Track and Field Sprint Start: A Systematic Review

**DOI:** 10.3390/ijerph19074074

**Published:** 2022-03-29

**Authors:** Maria João Valamatos, João M. Abrantes, Filomena Carnide, Maria-José Valamatos, Cristina P. Monteiro

**Affiliations:** 1 Sport and Health Department, Faculdade de Motricidade Humana, Universidade de Lisboa, Estrada da Costa, 1499-688 Cruz-Quebrada, Portugal; jabrantes@fpatletismo.pt (J.M.A.); fcarnide@fmh.ulisboa.pt (F.C.); mzvalamatos@fmh.ulisboa.pt (M.-J.V.); cmonteiro@fmh.ulisboa.pt (C.P.M.); 2 Interdisciplinary Center for the Study of Human Performance (CIPER), Faculdade de Motricidade Humana, Universidade de Lisboa, Estrada da Costa, 1499-688 Cruz-Quebrada, Portugal; 3 Biomechanics and Functional Morphology Laboratory, Faculdade Motricidade Humana, Universidade Lisboa, Estrada da Costa, 1499-688 Cruz-Quebrada, Portugal; 4 Neuromuscular Research Laboratory, Faculdade Motricidade Humana, Universidade Lisboa, Estrada da Costa, 1499-688 Cruz-Quebrada, Portugal; 5 Laboratory of Physiology and Biochemistry of Exercise, Faculdade de Motricidade Humana, Universidade de Lisboa, 1499-688 Cruz-Quebrada, Portugal

**Keywords:** track and field, sprinters, sprint start, block start, block velocity, biomechanics, kinematics, kinetics, sprint running, initial acceleration, sprint first stance, sprint first two steps

## Abstract

In athletics sprint events, the block start performance can be fundamental to the outcome of a race. This Systematic Review aims to identify biomechanical factors of critical importance to the block start and subsequent first two steps performance. A systematic search of relevant English-language articles was performed on three scientific databases (PubMed, SPORTDiscus, and Web of Science) to identify peer-reviewed articles published until June 2021. The keywords “Block Start”, “Track and Field”, “Sprint Running”, and “Kinetics and Kinematics” were paired with all possible combinations. Studies reporting biomechanical analysis of the block start and/or first two steps, with track and field sprinters and reporting PB100m were sought for inclusion and analysis. Thirty-six full-text articles were reviewed. Several biomechanical determinants of sprinters have been identified. In the “Set” position, an anthropometry-driven block setting facilitating the hip extension and a rear leg contribution should be encouraged. At the push-off, a rapid extension of both hips and greater force production seems to be important. After block exiting, shorter flight times and greater propulsive forces are the main features of best sprinters. This systematic review emphasizes important findings and recommendations that may be relevant for researchers and coaches. Future research should focus on upper limbs behavior and on the analysis of the training drills used to improve starting performance.

## 1. Introduction

The 100 m race is perhaps the highlight of the Olympic Games, as it defines who is the fastest man and woman in the world. In this type of event, the block start performance and the subsequent first two steps can be of critical importance since they have a direct influence on the overall 100 m time [1,2,3,4,5,6,7,8]. Given the importance of the sprint start, a new body of research has emerged in the past two decades that involved advanced technologies, high-precision methods, and sprinters of a higher performance level. For this reason, several technical (kinematic) and dynamic (kinetic) aspects are currently identified as determinant factors for starting block phase and initial sprint acceleration performances [1,4,6,9,10,11,12,13,14,15,16,17,18,19,20,21,22,23,24,25]. However, the concepts, outcomes, and findings between studies are sometimes inconsistent and difficult to interpret and conclude from. These inconsistencies may be accounted for by different study designs, methods, technologies of measure (e.g., external reaction forces under or on the blocks), statistical analyses, or more importantly, the ambiguity between samples of sprinters with different performance levels (e.g., elite, sub-elite, well-trained or trained) and/or between-group analyses based on the overall 100 m performance (i.e., personal best at 100 m—PB100m), and not on block performance. Although two important narrative reviews have already been published [26,27], to our knowledge, no previous review conducted a systematic search of literature exploring the inter-individual variability on block start performance across different performance levels. Thus, the main purposes of this systematic review were: (a) determine the biomechanical parameters of greatest influence on the sprint start, including the “set” position and push-off phase, and the first two steps of initial sprint acceleration and (b) identify the kinematic and kinetic biomechanical variables that best differentiate sprinters of different performance levels in each of those three phases of the sprint start. Considering the impact of the sprint in the sports field and the absence of systematic studies on the kinematics and kinetics factors that determine success in block starts and initial sprint acceleration, we hypothesized that this systematic review will have a relevant impact on researchers to better design experimental/intervention studies, as well as constituting relevant support for coaches and athletes in the definition of efficient strategies for performance in the 100 m race.

## 2. Materials and Methods

### 2.1. Article Search, Eligibility, Inclusion, and Exclusion Criteria

The systematic search of relevant articles was conducted based on PRISMA (Preferred Reporting Items for Systematic Reviews and Meta-analyses) guidelines [28]. PubMed, Web of Science, and SPORTDiscus databases were searched for the following mesh terms: “Block Start” OR “Track and Field” OR “Sprint Running” OR “Acceleration” AND “Kinetics and Kinematics” pairing them with all possible combinations. In addition, filters for ‘English’ and ‘articles’ have been applied. The last search took place on 30 June 2021.

The inclusion criteria were: publications in English; original observational and experimental studies published in peer-reviewed journals; studies mainly focused on the block phase and/or one or two of the subsequent stance phases concerning kinematic and kinetic variables; and studies that included track and field sprinters with the indication of their PB100m. The following types of records were excluded: conference abstracts; studies focused exclusively on the acceleration phase (beyond the first two stance phases) or mainly focused on limitations imposed by motor and neurological impairments; studies reporting data referring to samples evaluated in previously published papers; studies not mentioning the performance level of the sprinters through their PB100m; case reports; and studies without reference to biomechanical variables.

The records identified from the databases with the aforementioned mesh terms were exported to the reference manager software EndNote X8 that eliminated duplicates. All articles’ eligibility was then assessed independently by two reviewers’ authors (JMA and FC). The articles identified were first screened by title and abstract for relevance. Studies that raised any uncertainty in exclusion were conservatively retained for subsequent full-text review. The full text of the articles selected as relevant or having raised uncertainty in exclusion was read and further scrutinized for meeting the inclusion criteria and their quality was evaluated. Disagreements on final inclusion or exclusion of studies were resolved by consensus, and if disagreement persisted, a third reviewer (first author, MJV) was available for adjudication. Articles that did not meet the selection criteria or presented a quality score below 50% were excluded.

### 2.2. Quality of the Studies

The study quality of each publication was evaluated according to the guidelines of the Strengthening the Reporting of Observational Studies in Epidemiology (STROBE) Initiative [29]. This analysis was based on 22 items. Title and abstract. Introduction: background and rationale. Methods: study design, setting, participants, variables, data sources, bias, sample size, quantitative variables, and statistical methods. Results: participants, descriptive data, outcome data, main results, and other analyses. Discussion: key results, limitations, interpretation, and generalizability. Funding. These criteria were scored on a binary scale (1 = yes, 0 = no) independently by two of the authors, and a quality score was then calculated for each study by adding its binary scores and dividing the result by the maximum possible score the study could have achieved. This was then expressed as a percentage to reflect a measure of methodological quality. The quality scores were classified as follows (a) low methodological quality for scores < 50%; (b) good methodological quality for scores between 50% and 75%; and (c) excellent methodological quality for scores > 75%. The studies with a score lower than 50% [30] were excluded from the systematic review. The inter-rater reliability analysis was evaluated by the Cohen’s Kappa for nominal variables (2 dimensions) [31]. Standards for strength of agreement for the kappa coefficient were: ≤0 = poor, 0.01–0.20 = slight, 0.21–0.40 = fair, 0.41–0.60 = moderate, 0.61–0.80 = substantial, and 0.81–1 = almost perfect [32].

### 2.3. Data Extraction

An Excel form was used for data extraction. Of each manuscript selected for review, the following information was extracted from each included study: (a) the primary focus of study, means the phase of sprint start, e.g., block phase, first stance, and study design; (b) the main purpose, e.g., associations between biomechanical variables of starting blocks and the sprint start performance, comparing athletes of different performance levels, comparing different footplate spacing and block angles; (c) type of kinematic and kinetic analyses systems used—two dimensional (2D) or three dimensional (3D) analysis and starting blocks instrumented or placed on force platforms; (d) study sample—the number per gender of participants, and per level of expertise of participants according with the authors, and their PB100m; (e) biomechanical measurement protocols—the variables used to characterize the biomechanical factors of sprint start, number and distance of repeated trials; and (f) key findings of sprint start kinematic and kinetic factors.

## 3. Results

### 3.1. Search Results

The initial search identified 756 titles in the described databases. With the reference manager software, 406 duplicates were eliminated automatically. The remaining 350 articles were then screened according to title and abstract for relevance, resulting in another 289 studies being eliminated from the database. The full text of the remaining 61 articles was read and another 22 were rejected for not meeting the inclusion criteria defined for the current study and 3 studies were excluded for not meeting the quality criteria (quality index < 50%). A total of 36 studies was fully reviewed.

**Figure 1:**
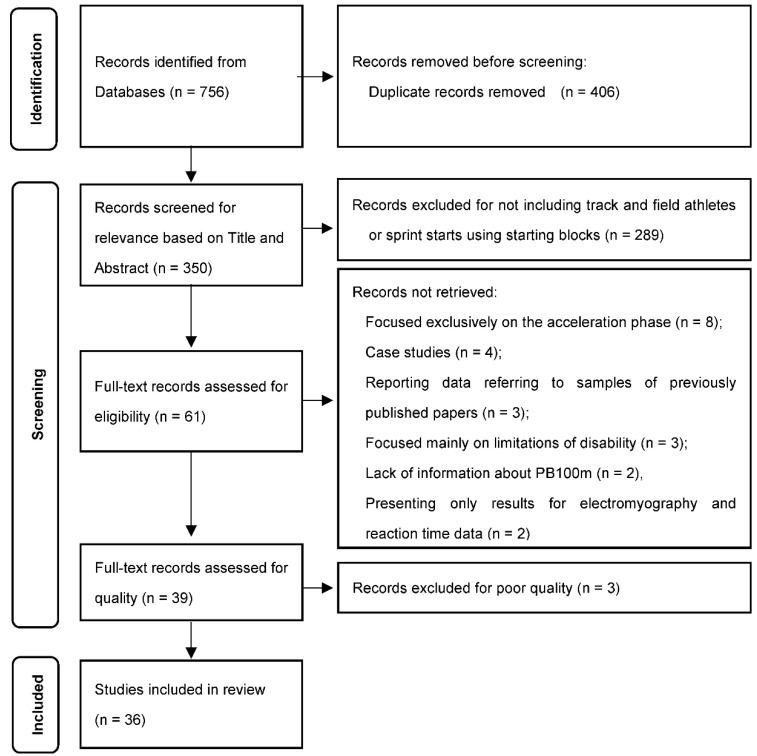
Flow diagram of the study selection process according to PRISMA guidelines.

Studies were excluded in the screening stage due to not including track and field athletes or sprint starts using starting blocks (n = 289). In the eligibility stage, there were several reasons for exclusion, namely studies with results focused exclusively on the acceleration phase (n = 8), case studies (n = 4), studies reporting data referring to samples of previously published papers (n = 3) or mainly focused on the limitations of disability (n = 3), lack of information about the PB100m (n = 2) and studies presenting only results for electromyography and reaction time data (n = 2). Figure 1 presents the complete flow diagram.

### 3.2. Quality of Studies

In the evaluation of methodological quality, the inter-rater reliability analysis achieved a Kappa value of 0.91 (0.84–0.98), indicating almost perfect agreement between raters. The mean quality score of the included studies was 74.92%. None of the studies achieved the maximum score of 100% and 3 studies (excluded) scored below 50%. Sixteen studies were classified with good methodological quality (quality score between 50 and 75%), while 20 studies had excellent methodological quality (quality score > 75%). The main deficiencies in methodological quality were related to the estimation of sample size and study limitations discussion.

### 3.3. Basic Characteristics of Included Studies

Fifteen studies [2,3,10,11,12,17,20,21,23,25,33,34,35,36,37] focused specifically on the block phase, 18 studies [1,4,5,6,7,8,13,14,15,16,18,19,24,38,39,40,41,42] on the block phase and, at least one of the subsequent two flight and stance phases, and 3 studies [9,22,43] on the initial acceleration (the first and/or the second step). A summary of all the individual studies reviewed is presented in Table 1.

Study purposes included evaluation of specific block start and initial acceleration variables and their influence on block performance (14 studies) [2,3,4,6,10,11,14,18,23,24,33,36,40,43]; analysis of different “set” position or block configurations (11 studies): location [20] and modulation [35] of center of pressure (COP) on the starting block surface, different block spacing [8,12,37] and widened conditions [21], different block plate obliquities [19,25,34], changed “set” position knee angles [41] and block pre-tension [17]; and comparisons between sprinters of different performance levels, despite the subjectivity associated with the descriptor of the performance level of the athletes (11 studies) [1,5,7,9,13,15,16,22,38,39,42]. The ambiguity in the performance level descriptors includes categories such as: elite vs. sub-elite or well-trained [7,16,22], world-class vs. elite [38], faster vs. slower [5], adult well-trained vs. trained [9,15,42]; elite or well-trained senior vs. junior academy, elite junior, U18 or young well-trained [1,39]; and top sprinters [13]. All studies comparing groups of athletes included male sprinters, but only 4 [1,9,15,38] included women of different performance levels. The studies included in the systematic review presented a cross-sectional study design, except for one study that presented a follow-up design [16]. 

Twenty-one studies evaluated kinetic variables from blocks start placed on force platforms (12 studies) [5,10,17,18,19,20,21,23,33,35,39,42] or instrumented starting blocks sensors (9 studies) [1,4,11,12,13,16,24,25,34]. Twelve studies [4,6,9,14,15,18,19,22,24,39,42,43] used a large variety of force platforms arrangements to analyze the dynamic characteristics of the first steps of the initial acceleration.

Concerning kinematic variables, a bi-dimensional analysis, including one or two high-speed digital cameras, was applied in 9 studies [3,12,13,18,19,25,34,37,40], and a 3D kinematic analysis, including 3 [38], 6 [16], or 8 or more cameras [5,6,7,8,9,21,24,36,41] was applied in 11 studies.

Total participants are 766 track and field sprinters, including 179 women and 587 men, and 11 non-trained male subjects [42]. Regarding the sample size of the individual studies selected, Chen, Wu [37] and Debaere, Delecluse [14] are those with the smallest number, 7 participants, and Schrodter, Bruggemann [25] conducted the study with 84 subjects (the largest sample size). The sample sizes from the other studies ranged from 8 [18,36] to 67 [1] subjects, with a mean sample size of 20 participants per study. The mean age of the participants in the selected studies ranged from 15.3 years (under 16) to 28 years. For women, PB100m ranged from 11.10 s (world-class) to 13.10 s (university level), with more classification terms being used, such as “elite” (11.29 to 11.95 s), “well-trained” (11.87 to 12.20 s), “trained” (<11.90 s), or “national level” (11.45 to 12.66) sprinters. Men were classified as “world-class” (10.03 to 10.98 s), “elite” (9.95 to 10.81 s), “sub-elite” (10.40 to 10.95 s), “well-trained” (10.65 to 11.77 s), “trained” (10.40 to 11.37 s), “national level” (10.58 to 11.22 s), “university level” (10.78 to 12.00 s), or just “sprinters” (10.50 to 11.24 s). Among studies, male PB100m ranged from 9.95 s to 12.00 s.

Through the analysis of the research setup protocols, it was possible to identify a “standard experimental setup”. Sixty-nine percent of the studies used distances between 10 and 30 m, with distances shorter than 10 m used only in 4 studies [5,24,41,43] and distances greater than 30 m used in 7 studies [10,20,22,33,37,38,39]. The number of trials performed ranged between 3 and 10 in 86% of the studies, but in 3 studies [10,20,38] the participants performed 1 or 2 trials, and in 2 studies [40,41] more than 10 trials. Fifty-eight percent of the studies were carried out on an indoor track, 4 studies [12,37,38,40] on an outdoor track, 2 studies [24,41] in a laboratory context, and 9 studies [1,8,10,16,20,23,25,42,44] did not mention the measurement location.

### 3.4. Data Organization and Analysis

There was a very large diversity of kinematic and kinetic variables reported among selected studies. Since it is impossible to discuss them all, we will highlight those reported as explicative of high levels of the sprint start performance and that best differentiate faster from slower sprinters. Based on the main findings highlighted in Table 1, the explanatory variables of superior performance levels were identified and systematized in a sequence of tables in Appendix A, Appendix B and Appendix C, related to the “Set” position (Appendix A Table A1), block phase (Appendix B Table A2 and Table A3), and first two steps of the initial acceleration (Appendix C Table A4 and Table A5). With this strategy of results presentation, it is expected that readers will have access to the primary data extracted from all the studies included in the systematic review. Therefore, Appendix A Table A1 summarizes the kinematic variables in the “Set” position, showing that anthropometry-driven block setting and muscle-tendon unit (MTU) length have an important role in the block start performance. Furthermore, faster sprinters tend to move their center of mass (CM) closer to the starting line and closer to the ground. Concerning joint angles, the knee angular position seems to be a greater performance predictor than any other lower limb joint. At the push-off phase (Appendix B Table A2 and Table A3, for kinematic and kinetic variables, respectively) a rear hip extension range of motion (ROM) and a rapid extension of both hips appear to be positively associated with block performance. Moreover, greater average force production during the push against the blocks, especially from the rear leg and particularly the hip, appears to be important for performance. A posterior COP location on block surfaces can also improve sprint performance. Immediately after exiting the blocks, shorter first flight durations and longer first stance durations (allowing more time to generate propulsive force) are the kinematic features of best sprinters (Table A4). During the first two steps of initial acceleration, higher levels of performance seem to be associated with shorter flight times, longer contact times, and the ability to extend the knee throughout both stance phases (Table A5).

## 4. Discussion

This paper systematically reviews the kinematic and kinetic biomechanical variables of the block start and initial sprint acceleration phase that influence performance and best differentiate sprinters of different levels. Despite the large number of variables reported in the reviewed studies it was possible to identify some that effectively best describe the influential factors of these events as they are associated with better performance outcomes or best differentiate sprinters of different performance levels. However, notice should be made to the difficulty in analyzing data between studies as there are still no standards for reporting the data, such as measurement units (e.g., m vs. cm) [12,17,18,35], joint angular measurement norms and conventions [3,4,6,12,13,36,38] and/or data normalization methodologies (e.g., for full-height/lower limb length, body mass/body weight) [2,4,17,22,24,25]. Additionally, there is some subjectivity associated with inconsistent descriptors of performance level [26], confirmed by the variability of the sprinter’s classifications used (e.g., from just sprinters to well-trained sprinters, elite sprinters, world-class sprinters, or high-level sprinters) [5,7,16,22,36,38,42]. Another critical factor that somehow may influence data variability between studies is the period of the season in which the data collection took place (e.g., prior to the competition phase of the indoor season vs. during the competitive indoor season or beginning of the summer season) [18].

To better understand the determinant factors of sprint start, the findings from the reviewed studies have been organized into three focuses: (i) the “set” position, (ii) the push-off phase, and (iii) the first two steps of initial acceleration, according to the data presented in Appendix A, Appendix B and Appendix C.

### 4.1. The “Set” Position

The “Set” position is the first performance key factor in the block start performance because it depends on block settings and the body posture assumed by sprinters. For the question: “Is there one optimal “Set” position which should be adopted by sprinters?” the answer seems to be no. The researched studies [3,38] showed that it is not an important differentiating factor of performance, since it does not present any correlation with PB100m or normalized block power [3]. However, there are some interesting aspects that sprinters should look out for in a more effective “Set” position [5,12]. The ideal “Set” position depends on the individual anthropometric features [12], strength [38], and morphologic characteristics and motor abilities [13].

#### 4.1.1. Block Settings

The “Set” position depends largely on the anteroposterior block distance, which defines the type of start used. There are three types of block starts based on inter-block spacing: bunched—less than 0.30 m; medium—0.30 to 0.50 m; and elongated—greater than 0.50 m [27,37].

Studies that reported block spacing based on the individual sprinter’s preferences [5,12,13,18,35] reported distances between 23.5 ± 1.9 cm (for female sprinters; PB100: 11.97 ± 2.6 s) [13] and 32 ± 5 cm (for male sprinters; PB100m: 10.79 ± 0.21) [18]. This suggests that most sprinters adopt distances within or very close to the bunched start type, favoring CM positioning closer to the starting line [7,38]. Slawinski, Dumas [8] have demonstrated that elongated start settings increase the block velocity (i.e., horizontal CM velocity at the block clearing [7]), but linked to an increase in the pushing time on the blocks which implies a significantly worse performance at 5 and 10 m compared to the bunched start. The same authors showed that the medium start offers the best compromise between the pushing time and the force exerted on the blocks, allowing better times at 10 m [8]. Additionally, more recently, Cavedon, Sandri [12] have demonstrated that the anthropometry-driven block setting based on the sprinter’s leg length has an important role in the block start performance leading to a postural adaptation that promotes several kinematic and kinetic advantages [12]. Adjusting inter-block spacing to the relative lengths of the sprinter’s trunk and lower limbs (increasing 25.02% the usually bunched start inter-block spacing), allows greater force and impulse on the rear leg and greater total normalized average horizontal external power (NAHEP) [12], the latter one identified as the best descriptor of starting block performance [2].

Other blocks setting features that should be considered in the “set” position are the feet plate obliquity and the amount of pre-tension exerted on the blocks prior to the gunshot. The block inclination (relative to the track) affects the plantar flexor muscle-tendon units’ (MTU) initial lengths and determines the muscle mechanics and the external force parameters during the block phase [19,25,34]. Faster sprinters presumably produce the peak torque at longer MTU lengths and adopting a more crouched position would allow them to produce a higher force on the block phase [38]. Research data shows that reductions in both footplates’ inclinations (from 65 to 40°), meaning more muscle-tendon pre-stretch, lead to acute increases in block velocity and higher peak joint moments and powers, especially in the ankle [19]. Reductions in front block inclination alone (from 70 to 30°) also acutely increase block velocity without affecting push-off phase duration [34]. In another study [25], however, a greater mean rear block horizontal force was achieved by switching the rear foot to a steeper position (to 65°). This potential conflict between evidence might have arisen from differences in the location of the COP and the length of the footplates’ surface between studies since a better sprint start performance is accomplished with a higher and more to the rear COP on the starting block surface [20,35]. Conversely, a pre-tensioned start does not seem to yield a performance advantage over a conventional start, because the increase in the propulsive force of the lower limbs is reversed by an increase in the back force exerted through the hands during the same period [17].

#### 4.1.2. Sprinter Body Posture

Apart from block configuration, the choice of the sprinter’s body posture also determines the effectiveness of the “Set” position on the subsequent block push-off phase. The horizontal distance between starting line and the vertical projection of the CM to the ground in the “Set” position (XCM) [7] is a factor that differentiates sprinters with different performance levels. As said before, faster sprinters tend to move their CM closer to the starting line [7,38] and closer to the ground [38]. Elite (PB100: 10.27 ± 0.14 s) and well-trained (PB100: 11.31 ± 0.28 s) male sprinters showed XCM of 22.9 and 27.8 cm, respectively [7]. Likewise, world-class (PB100: 11.10 ± 0.17 s) and elite (PB100: 11.95 ± 0.24 s) female sprinters presented XCM of 16.2 and 24.8 cm, respectively [38]. This more crouched position is only possible due to the high explosive strength of best sprinters, which allows them to produce higher levels of strength in the blocks [38] and reduce the horizontal travel distance of the CM. This body position is complemented by a more advanced shoulder position, putting more tension on the arms, allowing greater blocking speed during the subsequent phase [7].

Related to sprinter joint angles configuration in the “set” position, Milanese and Bertucco [41] have shown that horizontal CM velocity at the block take-off and along the first two steps increases significantly when the rear knee angle is set to 90° instead of 135° or 115°. A 90° rear knee angle allows for a better push-off of the rear leg than larger angles, showing such condition may be a strategy that allows some elite sprinters to maximize their strength capacity [41]. A more flexed front knee may facilitate the optimal joint moment production, but only in sprinters with exceptionally high levels of explosive strength [38].

### 4.2. The Push-Off Phase

The “block-phase” or “push-off phase” in the starting blocks initiates immediately after the gunshot and is considered a complex motor task that helps to determine sprint start performance [1]. Reaction time is the first factor in the time sequence of the block phase and it is the period from the gun signal to the first measurable change of pressure detected in the instrumented blocks [16]. While a sprinter’s ability to react is undeniably important, it is related to the information-processing mechanisms that do not seem to correlate with the performance level [7,45] and, therefore, is beyond the scope of our review (for a review of factors that affect response times, see Milloz, Hayes [46]). Having reacted, the aim of the block phase is to maximize horizontal velocity in as little time as possible. The motion variables during the block phase are, therefore, the focus of this section.

#### 4.2.1. Push-Off Kinematics Analysis

The efficiency of the starting action depends mainly on the compromise between horizontal start velocity (or block velocity) and the block time (referring to the time elapsing from the first movement at the “set” position to the exiting from the block [7]), resulting in the horizontal start acceleration [13]. Despite the horizontal block velocity could be considered the main parameter for an efficient sprint start [13], it cannot be used solely [2] because an increased block velocity could be due to either an increase in the net propulsion force generated or to an increased push-off duration [2,18]. Thus, best sprinters tend to present higher block velocity and greater block acceleration than slower sprinters [1,5,7,13,16,22,39,42], because they are able to produce a greater impulse in a shorter time [2,5,36] and optimize their force production on the blocks [16,19]. In fact, if sprinters increase their anteroposterior force impulse (FI = force × time) from a longer block time, they decrease their block acceleration [2,42] and the performance at 5 and 10 m [8]. Studies comparing data between sprinters of different performance levels mostly show higher block velocities (3.38 ± 0.10 vs. 3.19 ± 0.19 m·s^−1^; 3.48 ± 0.05 vs. 3.24 ± 0.18 m·s^−1^; 3.61 ± 0.08 vs. 3.17 ± 0.19 m·s^−1^; and 3.36 ± 0.15 vs. 3.16 ± 0.18 m·s^−1^) [5,7,22,33] and greater block accelerations (9.5 vs. 8.8 m·s^−2^; 8.2 vs. 7.9 m·s^−2^; 9.72 vs. 8.4 m·s^−2^; and 7.47 vs. 7.35 m·s^−2^) [1,5,7,42] for faster sprinters. Furthermore, higher performance levels also appear to be slightly related to lower block vertical velocities [38] and more horizontal CM projection angles (i.e., resultant direction from the CM horizontal and vertical block exit velocities) [33,39].

Lower limbs joints pattern during the pushing phase (i.e., from movement onset until block exit) is mostly associated with extension movements, especially on the hips and knees [3,4,6,25,36]. The front leg joints typically extend through a considerable ROM in a proximal-to-distal extension pattern [3], reaching their maximum at the beginning of the flight phase (e.g., hip: 183.2 ± 6.8°, knee: 177.4 ± 5.2°, and ankle: 133.1 ± 6.7°) [6]. Contrarily, the rear leg does not exhibit the same proximal-to-distal extension strategy, with the knee reaching its peak angular velocity before the hip and the ankle [3,36]. This happens perhaps due to considerably less ROM of the rear knee compared to the front knee [3], as it starts from a more extended angle in the “set” position (e.g., rear knee: 120.7 ± 9.7°; front knee: 91.0 ± 9.8°). The movement of the ankles is more complex because it involves first a dorsiflexion and after an extension resulting in a stretch-shortening cycle of the triceps surae muscle [3,6,25,36]. The duration of the ankle’s flexion is greater for the rear ankle (50% of the block phase) than for the front ankle (20% of the block phase) [36]. Experimental manipulations on footplates’ inclinations [19,34] have shown an inverse association between block angles and muscle-tendon lengths of the gastrocnemius and soleus, highlighting that block angles steeper than 65° could have disadvantageous effects on plantar flexor function [19]. Peak angular velocities at both hips are reached by a combination of flexion–extension, abduction–adduction, and internal–external rotation [23,36], reinforcing the importance of a 3D analysis of the sprint start [36]. Whilst there is a consistent trend among sprinters in the joint angular velocity sequence during the block phase, the lack of comparative data between sprinters of different performance levels does not allow to highlight the technical aspects critical to success. However, a rapid hip extension should be one of the first aspects to consider on a sprinter’s technique during the start, as peak angular velocities at both hips and rear hip range of extension are positively associated with block power (*r* = 0.49) [3].

Although upper body kinematics in the push-off phase has been the focus of a small number of studies, some important findings are noteworthy. The action of the upper limbs is more variable between sprinters than that observed for the lower limbs [36]. Despite this, it is possible to recognize a 3D movement pattern for shoulders and trunk with a combination of flexion–extension, abduction–adduction, and internal–external rotation movements, while the elbows exhibit an extension and pronation movement [36]. The velocity of the rear shoulder tends to be slightly greater than that of the other joints, but the peak resultant angular velocities at the upper limb joints are comparable to those at lower limbs during the push-off phase, particularly that of both knees and front ankle [36]. However, there is no evidence linking different upper limb kinematic patterns with any block phase performance predictor, and further research is needed to compile relevant recommendations for athletes and coaches.

#### 4.2.2. Push-Off Kinetic Analysis

According to Newton’s second law of motion, horizontal CM acceleration requires net propulsive forces to be applied to the athlete’s body in the sprinting direction. Therefore, as said before, the horizontal force impulse, made up by the mean horizontal force and push-off time, is the determining factor of the horizontal velocity at block exit [2,5,36,42]. The relationship between these factors (i.e., horizontal force and push-off time) shows that the application of a greater amount of horizontal force is a key performance factor [42], as an increase in the time action (block time) conflicts with the criterion for 100 m performance: ‘shortest time possible’. Thus, best sprinters generate greater average forces [10,22], higher rates of force development [7,25], and larger net [7] and horizontal [5] block impulses than their slower counterparts. Likewise, Graham-Smith, Colyer [39] comparing senior to junior athletes also showed that sprinters with faster PB100m (senior athletes) exhibit higher relative horizontal force during the initial block phase and higher forces during the transition from bilateral to unilateral pushing [39]. The evident importance of the force generated against the blocks for proficient execution of the starting block phase has encouraged researchers to gain a deeper understanding of the kinetic determinants of such a crucial phase of sprinting. Bezodis, Salo [2] tried to find the push-off performance measure that was more adequate, objective, and possible to quantify in the field. From their analysis, the NAHEP was identified as the most appropriate measure of performance because it objectively reflects, in a single measure, how much sprinters are able to increase their velocities and the associated length of time taken to achieve this, whilst accounting for variations in morphologies between sprinters [2]. Later, the identification of the magnitude of the force applied to both blocks and their optimal orientation as major determinants of performance encouraged researchers to gain a deeper understanding of the push-off forces applied against each block separately. Consequently, some studies support the importance of the force generated by the front leg for forwards propulsion [6,42] and show that faster sprinters are able to produce higher force impulses in the front block than slower sprinters [5,33] (for example: 221.3 ± 15.8 N·s vs. 178.3 ± 13.1 N·s for faster and slower sprinters, respectively [5]). Colyer, Graham-Smith [33] reinforce this feature highlighting that higher front block force production during the transition (when the rear foot leaves the block, 54% of the block push) and a more horizontally orientated front block force vector in the block phase (81–92%) are important performance-differentiating factors. However, other evidence ensures that the rear block force magnitudes are the most predictive external kinetic features of block power [10,33] and sprint performance [5,7,12,16]. For example, Coh, Peharec [5] found that a faster group of sprinters (PB100m = 10.66 ± 0.18 s; 913 ± 89.23 N) produced greater total forces against the rear block than a group of slower sprinters (PB100m = 11.00 ± 0.06 s; 771 ± 55.09 N). A longer relative rear leg push (i.e., as a percentage of the total push-off phase) is also positively associated (*r* = 0.53 [3]) with greater block power [3,10] and is present in sprinters with faster PB100m [5,7,33]. Modulations of the COP on the starting block surface showed that COP location may also be related to initial sprint performance [20,35]. Better sprint start performance appears to be achieved with a higher and more to the rear COP during the force production phase [20]. Thus, athletes and coaches should keep in mind that pushing the calcaneus onto the block (posterior location) may improve the 10 m time and/or horizontal external power for some individuals [35].

Forces under the hands have been reported in relatively few studies [10,33,42], showing somewhat contradictory results. While some point to a primary support role [42], others point out that the best athletes produced less negative horizontal impulse under hands compared with their slower counterparts [33]. Therefore, the importance of the hands’ kinetics during the push-off phase remains unclear and should be the subject of future research.

In addition to external kinetic analyses, which provide valuable insight into starting block performance, the analysis of internal kinetics (i.e., joint kinetics) helps to increase the understanding of the segment motions that are responsible for CM acceleration. Recent research of joint kinetics has shown that 55% of the variance in NAHEP of a group of sprinters with a PB100m of 10.67 s was mainly accounted for by rear ankle joint moment (23%), front hip joint moment (15%), and front knee joint power (15%). The remaining 2% was shared by the remaining lower limbs joint kinetic variables [11]. In the rear block, the magnitude of the horizontal force produced is determined by the rear hip extensor moment and the rear hip extensor power coupled with large ankle joint plantarflexion moment [4,11,19], without any significant knee joint contribution [4,11]. At the front block, a proximal–distal pattern of peak joint power is evident [4], highlighting a strategy often adopted in power demanding tasks, with the main periods of positive extensor power at the front ankle and knee occurring after the rear foot has left the block [4]. In a study with 12 sprinters from the University of Tokyo team (PB100m: 10.78 ± 0.19 s), Sado, Yoshioka [23] showed that the peak lumbosacral extension moment was significantly larger than any other lumbosacral and lower-limb moment, being positively correlated with the starting performance. This peak value appeared in the double-stance phase where both hip joints exerted extension moments. The aforementioned evidence supports the findings of Slawinski, Bonnefoy [36] who showed that the lower limbs and the head–trunk segments are the two main segments that contribute to the kinetic energy of the total body. Upper limbs contribute 22% to the total body kinetic energy, demonstrating that their actions in the pushing phase on the blocks are not negligible [36].

### 4.3. The First Two Steps

The primary goal of the first steps is to generate a high horizontal velocity [40]. However, the transition between block start and the first steps represents a specific biomechanical paradigm: integrate temporal and spatial acyclic movements into a cyclic action [5]. The efficiency of this transition depends on the biomechanical demands of the first stances after block clearance, which are very different from the other stances during acceleration [14]. The sprinter aims to generate maximal forward acceleration during the transition from start block into sprint running [2,14,22,42] while generating sufficient upward acceleration to erect itself from a flexed position in the start blocks to a more extended position [6,14]. Specific technical (kinematic) and dynamic (kinetic) skills are therefore needed to successfully achieve this transition, and they are the focus of this section.

#### 4.3.1. First Two Steps Kinematic Analysis

The primary goal of the initial steps of a sprint running is to generate a high horizontal sprint velocity, which results from the product of the length and frequency of the sprinter’s steps [22,40]. Spatiotemporal parameters have shown that the sprinter’s step length increases regularly during the acceleration phase, while step frequency is almost instantaneously leveled to the maximum possible [22]. Typically, the step frequency reaches the maximal values very quickly (80% at the first step and about 90% after the third step) [22], achieving around 4 Hz immediately after block exit [26,40]. The length of the first steps is more variable between sprinters, ranging from 0.82 to 1.068 m (senior females) [1,38] or 0.85 to 1.371 m (senior males) [1,7] on the first step, and from 1.06 to 1.30 m (senior females) [1,13] or 1.053 to 2.10 m (senior males) [7,37] on the second step. Despite this variability, step length tends to be longer in faster sprinters, particularly in the first step (e.g., 1.371 ± 0.090 vs. 1.208 ± 0.087 m [7]; 1.30 ± 0.51 vs. 1.06 ± 0.60 m [5]; 1.135 ± 0.025 vs. 0.968 ± 0.162 m [38]), exhibiting an increase of about 14 cm for every 1 s less in PB100m [38]. This may be a consequence of the lower vertical velocity of the CM at the block clearing shown by faster sprinters, allowing them to travel a longer distance despite shorter flight times [38]. Indeed, the kinematics of faster sprinters is also characterized by a tendency to assume long ground contact times in the first two steps (e.g., mean first contact duration for Diamond League sprinters is 0.210 s for males and 0.225 s for females, which is greater than those of lower-level Italian junior sprinters: 0.176 and 0.166 s, respectively), associated to short flight times (0.045 and 0.064 s, for the first flight of world-class and elite male sprinters, respectively) [38]. This strategy allows the high-level sprinters to optimize the time during which propulsive force can be generated, minimizing the time spent in flight where force cannot be generated. Combined with this, best sprinters have their CM projected further forward [7] at the first touchdown, putting the foot behind the vertical projection of the CM [3], and minimizing the braking phase. At the takeoff of the first and second steps, the CM horizontal position is also greater in elite than well-trained sprinters [7]. This means that the CM resultant and horizontal velocity in the first two steps are generally greater in high-level sprinters [7,15]. Slawinski, Bonnefoy [7], for example, reported that elite sprinters have a CM resultant velocity 5.8% higher than well-trained sprinters, at the end of the first step (4.69 ± 0.15 vs. 4.42 ± 0.11 m·s^−1^ for elite and well-trained sprinters, respectively). Furthermore, high-level sprinters also show slightly lower vertical velocities [7,39] and more horizontal CM projection angles at the end of the first two support phases [39].

Lower limb joints pattern during the first two steps is associated with a proximal-to-distal sequence of the hip, knee, and ankle of the stance leg [4,9,43]. During both first and second steps, the ankle joint undergoes dorsiflexion during the first half of stance (e.g., 17 ± 3° and 18 ± 3° for the first and second steps, respectively [43]) and subsequently a plantarflexion movement (e.g., 45 ± 6° and 44 ± 5° for the first and second steps, respectively [43]).

The hip performs extension for the entire stances, the knee extends until the final 5% of stances, and the ankle is dorsi-flexed during the first half of stances before the plantar flexing action [6]. After leaving the rear block, there is a small increase in ankle joint dorsiflexion during the swing phase, preceding the plantarflexion that occurs just before touchdown [6]. Although the ankle plantar-flexes slightly at the end of the flight, the ankle is in a dorsi-flexed position at initial contact (e.g., first stance: 70.6 ± 5.8° and second stance: 72.4 ± 7.1° [6]). During both first and second steps, the ankle joint dorsi-flexes during the first half of stance (e.g., 17 ± 3° and 18 ± 3° for the first and second steps, respectively [43]) and subsequently performs a plantarflexion movement (e.g., 45 ± 6° and 44 ± 5° for the first and second stance, respectively [43]). Note that a reduction in the range of dorsiflexion during early stance, requiring high plantar flexor moments, has already been associated with increases in first stance power [47]. Maximal plantarflexion occurs immediately following takeoff reaching, for example, 111.3° at the first stance and 107.1° at the second stance [6]. The extension of both knees occurs just after the block exit and reaches its maximum at the beginning of the flight phase, with larger extension in the front compared with the rear leg (e.g., rear: 134.9 ± 11.2°; front: 177.4 ± 5.2°) [6]. From a flexed position at initial contact, the knee extensors generate power to induce extension throughout stance and to attain maximal extension at takeoff, achieving peak extension angles of around 160–170° (not full extension; e.g., first stance: 165.2 ± 20.6°; second stance: 163.6 ± 17.7° [6]). This extension action of the knee during stances on its own may play a role in the rise of the CM during early acceleration [26]. The hip joints extend during block clearance to reach maximal extension during the beginning of the flight phase. During stance, the hips are in a flexed position at initial contact and continue to extend throughout stance, achieving maximal extension immediately following takeoff (e.g., first stance: 180.6 ± 20.9°; second stance: 181.1 ± 20.0° [6]). There is also a considerable ROM in hip and pelvis rotation during stance as well as abduction. Although there are detailed descriptions of the lower limb angular kinematics during the first two stances and flight phases [3,6], there seems to be no clear evidence about the joint kinematic features that differentiate faster from slower sprinters. Furthermore, there is also a lack of experimental data on arm actions during early acceleration and its relationship to performance descriptors, making necessary future research in this area to help identify the most important performance features.

#### 4.3.2. First Two Steps Kinetic Analysis

As said before, fast acceleration is a crucial determinant of performance in sprint running, where a high horizontal force impulse in a short time [13] is essential to reach high horizontal velocity [43]. Thus, as the highest CM acceleration during a sprint occurs during the first stances [7,9,14] (e.g., first stance: 0.36 ± 0.05 m·s^−2^; second stance: 0.23 ± 0.04 m·s^−2^ [14]), the ability to generate during this phase greater absolute impulse [7,18], maximal external power [39,42], and a forward-leaning force oriented in the sagittal plane [21,22,24,42] is linked to an overall higher sprint performance. Larger propulsive horizontal forces are particularly important during early acceleration, being a discriminating factor for superior levels of performance [48]. Experienced male sprinters (PB100m: 10.79 ± 0.21 s) can produce propulsive horizontal forces of around 1.1 bodyweight during the first stance [18]. However, a negative horizontal force has also been reported during the first contact after the block exit, even if the foot is properly placed behind the vertical projection of the CM [18]. During the first stance, for example, the braking phase represents about 13% of the total stance phase and the magnitude of the braking forces can reach up to 40% of the respective propulsive forces [18].

Furthermore, 3D analysis studies also highlight a lower body motion outside the sagittal plane during the first few ground contact phases [6,21,22,24,36,42]. In fact, during the first steps of a sprinter, a stance medial deviation is often observed that results from an impulse in the transverse plane. Although the medial impulse is the smallest of the three orthogonal stance impulses [21,22,42], the fact that it is non-zero can have an effect on the motion of the CM and on step width. However, it has been shown that well-trained sprinters present similar step widths in the early acceleration to those of the trained and non-trained sprinters [42]. Moreover, manipulations of both “set” position [21] and first step [24] widths have shown no effect on block-induced power nor braking force or net anteroposterior impulse, showing that smaller step width is not a discriminator factor of superior performance levels. Therefore, the perception that the adoption of a widened stance during initial acceleration (referred to as “skating style”) is detrimental to performance is not at all proven, and further research is needed to clarify the joint and muscular factors that contribute to the sprinters’ lateral motion in the initial phase of acceleration.

At joint level, the hip, knee, and ankle joints generate energy during stance leg extension [6], although it appears that the ankle joint is the main contributor to CM acceleration [14]. However, experimental and simulation studies highlight that the knee plays an important role during the first stance, being decisive for forward and upward CM acceleration [4,6,14,15]. The importance of power generation at the knee seems to be specific for the first stance when the knee is in a more flexed position and the sprinter is leaning forward. From the second stance onwards, the knee becomes less and the ankle more dominant since the plantar flexors are in a better position to contribute to forward progression [6]. As the knee is in a flexed position during the first step, the sprinter favors the immediate power generation of the knee extensors rather than preserving a stretch-shortening cycle [6]. In contrast, a stretch-shortening mechanism can be confirmed at the hip and ankle [4,6,14,15]. Hip extensors maximal power generation occurs near touchdown [4,6] where the hip extensors actively pull the body over the touchdown point [6]. The hip can effectively generate large joint moments and power [14], but only contributes minimally to propulsion and body lift during the first two stances [14]. Ankle plantar flexors act throughout both the first and second stances under a stretch-shortening cycle. There is therefore an initial phase of power absorption preceding the forceful power generation at take-off [4,14]. As a major contributor to CM acceleration, the ankle joint can generate up to four times more power than it absorbs during the first two stances [43]. Nevertheless, the importance of ankle stiffness during the first two stances remains unclear. While Charalambous, Irwin [49], in a case report, found a correlation between greater ankle stiffness and greater horizontal CM velocity at take-off (*r* = 0.74), Aeles, Jonkers [9] did not, still highlighting the lack of differences between faster (senior) and slower (junior) sprinters. Future work is therefore needed to further clarify this issue. Furthermore, it remains unclear whether ankle stiffness is influenced by foot structure and function (e.g., planus, rectus cavus, clubfoot) as well as other important performance variables such as greater maximal power, a forward-leaning force oriented in the sagittal plane, or COP location during push-off.

Concerning kinetic factors differentiating senior and junior athletes, Graham-Smith, Colyer [39] reported that, contrarily to the block phase where there are marked differences between groups, the force and power waveforms relating to the first two steps did not differ considerably across groups. Still, senior sprinters are able to produce greater horizontal power during the initial part (10–19% of the stance phase) of the first and second ground contact (first step: 25.1 ± 3.6 W·kg^−1^ vs. 23.1 ± 6 W·kg^−1^ and second step: 26.7 ± 3.6 W·kg^−1^ vs. 24.9 ± 4.5 W·kg^−1^, for senior and junior sprinters, respectively), and also exhibit a higher proportion of forces immediately after braking forces are reversed (from 9% to 15% and 25% to 29% of stance phase) [39]. Furthermore, Debaere, Vanwanseele [15] also highlight that adult sprinters are able to generate more joint power at the knee during the first step compared to young sprinters, inducing longer step length and therefore higher velocity [15]. Younger sprinters tend to prioritize a different technique: the hip contributes more to total power generation, while the knee contributes far less [15]. This indicates that younger sprinters lack the specific technical skills observed in adult sprinters, likely due to less musculature than adults [1,9,15]. However, there is no evidence of differences in ankle joint stiffness, range of dorsiflexion, or plantar flexor moment between young and adult sprinters [9]. This indicates that the technical performance-related parameters of the first stances are not likely to explain the better 100 m sprint times in adult compared to young sprinters [9].

### 4.4. Strengths, Limitations, and Recommendations

A strength of this review was that it allowed us to identify a body of knowledge that provides fundamental information for athletes and coaches as relevant data that can contribute to improving the training and/or preparation strategies for better performance, supported by scientific evidence.

A possible limitation of this systematic review is that it only includes studies written in English, thereby potentially overlooking other relevant publications in other languages. Additionally, the present article reviewed only studies with mention to sprinters’ PB100m, eventually precluding publications with relevant samples that could also add knowledge. Furthermore, extending the biomechanical analysis to muscular features beyond the simple kinematic and kinetic approach might have allowed a further understanding of the discriminating factors of superior performance levels. Another obvious limitation is the limited amount of research with female sprinters. Indeed, in the reviewed studies, there is a clear imbalance between the amount of female and male sprinters included (179 females vs. 587 males), questioning whether the biomechanical characteristics of the sprint start previously associated to female sprinters are attributable to sex-related aspects, or, rather, to aspects related to the 100 m time. Moreover, some of the studies included in this review were based on a relatively small sample size, especially when elite or world-class sprinters were included. This problem reflects the difficult access to high-level athletes, preventing the clear identification of discriminatory factors of superior performance levels. Finally, the conflicting classifications of sprinters level and the scarcity of information on effectively high-level or world-class sprinters, makes it difficult to compare sprinters of different performance levels. Considering entry standards for 100 m sprint event at the 2022 European Athletics Championships (10.16 s for men and 11.24 s for women), it can be said that a very small percentage of elite and/or world-class sprinters [50] was included in the reviewed studies.

Research on the biomechanics of the block and/or first stance phases has been the subject of growing interest in the past few years. Nonetheless, there are some unclear features in the studies published so far, which should be investigated in future studies for a better understanding of: (i) the association between different upper limb patterns and the main block start performance predictors; (ii) the influence of foot type (e.g., planus, rectus cavus, clubfoot on sprint start performance; (iii) the association between ankle stiffness during dorsiflexion and the horizontal CM velocity at take-off; (iv) the specificity characteristics of training drills, utilizing temporal organization and intra-limb joint coordination analyses, to help the process of exercise selection to enhance block starting performance; (v) how technical and/or physical training can improve ankle and knee function during first steps and increase horizontal velocity in the early acceleration; (vi) the influence of sex (such as physical or muscle structures and/or anthropometric characteristics) on sprint start performance descriptors. A major challenge for researchers is to align these research lines with the need for greater information on world-class sprinters during competition. Whenever possible, research based on a marker-less methodology and obtained during official top-level sprint competitions, during which the sprinters are supposedly more motivated to produce their best performance, should be encouraged.

It is worth mentioning two new studies [51,52] published after the date of this systematic review, which, meeting the defined inclusion criteria, could have added important knowledge on some of the issues mentioned above.

## 5. Conclusions

Based on this review, some important conclusions and recommendations to help athletes and coaches can be made, namely: (i) the choice of an anteroposterior block distance relative to the sprinter’s leg length may be beneficial for some individuals, promoting greater block start performance (greater normalized average horizontal external power); (ii) the use of footplate inclinations that individually facilitate initial dorsiflexion should be encouraged—footplate angles around the 40° are recommended and block angles steeper than 65° should be avoided; (iii) pushing the calcaneus onto the block (posterior location) may be beneficial for some individuals, improving the 10 m time and/or horizontal external power; (iv) short block exit flight times and optimized first stance contact times should be encouraged, as they maximize the time during which propulsive force can be generated; (v) focus attention on the magnitude of force applied on the rear block, as it is considered to be a primary determinant of block clearance; (vi) rapid hip extension during the push-off phase should be a priority in sprinter focus and coach feedback; (vii) the large role played by the hips on the push-off phase and by both the knee and ankle at the early stance must be acknowledged within physical and technical training to ensure strength and power are developed effectively for the nature of the sprint start.

## Figures and Tables

**Table 1:** Studies are listed in reverse-chronological order by year, followed by alphabetically for studies published in the same year. Samples (n) are restricted to total participating sprinters and are classified by performance level(s) according to the original authors.

Study Details				Sample				Main Findings	Quality
Reference	Primary Focus of the Study	Main Purpose	Biomechanics Analysis	Sex	n	Level	PB100m (s)		Mean Score (%)
Werkhausen, Willwacher [43]	First 2 steps. Two force platforms for the GRFs of the first 2 steps. Three-dimensional kinematic model (pelvis and lower limbs)	Investigate how plantar flexor muscle-tendon behavior is modulated during the first 2 steps	3D GRF of the first 2 steps	F	11	Germany national level	12.66 ± 0.49	Ankle and knee joint angles revealed no statistical differences at any time of both steps. Ankle joint power was negative after touchdown and positive during the rest of the stance phase, whereas net ankle joint work was positive during both steps. Knee joint power was positive during most of the stance phase.	67.78
Graham-Smith, Colyer [39]	Block phase and first 2 steps. An array of 6 force platforms.	Compare force production between elite senior and junior academy sprinters	3D block and first 2 steps GRF, and spatiotemporal data	M M	17 20	Elite Senior Junior Academy	8.2% worse than senior WR ^(a)^ 12.2% worse than junior WR ^(b)^	Senior sprinters presented higher relative anteroposterior force and power during the initial block phase, higher forces during the transition from bilateral to unilateral pushing and lower (more horizontal) projection angle across the initial 2 steps of the sprint compared with junior athletes.	76.82
Nagahara, Gleadhill [35]	Block phase. Two force platforms with a coordinate transformation matrix to the coordinate block system.	Examine whether modulation of COP location on the starting block improves sprint start performance	3D GRF under each block and spatiotemporal data	M	20	National level	11.22 ± 0.41	The modulation of COP location did not show an effect on AHEP and 10 m time. However, instructing to push the calcaneus onto the block (posterior location) may improve the 10 m time and/or AHEP for some individuals and may be accomplished through a shorter reaction time.	82.50
Sado, Yoshioka [23]	Block phase. Separated starting blocks secured onto separate force plates.	Examine the 3D lumbo-pelvic-hip kinetics during block start	3D GRF under each block	M	12	University of Tokyo team	10.78 ± 0.19	The peak lumbosacral extension torque was larger than any other peak torque.	66.36
Bezodis, Walton [10]	Block phase. Four synchronized force platforms under each block and each hand.	Identify the continuous GRF features which contribute to blocking phase performance	3D GRF under each block and each hand	M	23	Trained	11.37 ± 0.37	The resultant magnitude of the GRFs on the rear block is the most important predictor of block phase performance, followed in importance by front block force magnitude features. Features related to the direction of application of these forces are not relevant predictors of performance	87.50
Cavedon, Sandri [12]	Block phase. Instrumented starting blocks and 2 high-speed video cameras.	Analyze the effect of 2 block setting conditions on block start performance	2D kinematic, horizontal, and vertical forces components, and spatiotemporal data	M ^(c)^ F	22 20	Regional and National	10.45–11.30 11.45–12.68	An anthropometry-driven block setting condition based on the sprinter’s leg length was associated with several significant changes in postural parameters at the “set” position, as well as in kinetic and kinematic variables at the pushing and acceleration phases in comparison with the sprinter’s usual block setting, leading to improved performance	88.64
Colyer, Graham-Smith [33]	Block phase. Four force platforms under each of the legs and arms separately.	Analyze the associations between block reaction forces and average horizontal external power	2D anteroposterior and vertical block reaction forces, and spatiotemporal data	M	5 32 20	Elite National ^(d)^ Academy	<10.15 --- ---	Both higher magnitudes of force and more horizontally orientated force vectors were associated with higher performance levels. The ability to sustain high forces during the transition from bilateral to unilateral pushing was a performance-differentiating factor. Faster sprinters produced less negative horizontal impulses under hands compared with their slower counterparts	78.60
Nagahara and Ohshima [20]	Block phase. Two force platforms under each block.	Examine the association of block clearance performance with COP location on the starting block surface	3D GRF under each block	M	21	Sprinters	11.24 ± 0.41	The COP location was related to sprint start performance (AHEP). Better sprint start performance was achieved with a higher and more to the rear COP on the starting block surface during the force production phase	75.45
Sandamas, Gutierrez-Farewik [24]	Block phase and 1st stance. Three-dimensional kinematic full-body model. Instrumented blocks and a force platform. Natural technique (Skating); 1st step inside a 0.3 m lane (Narrow).	Analyze the block reaction forces when 1st step width is manipulated	3D kinematic data and external block and 1st step reaction forces	M F ^(e)^	8 2	Competitive, Including international championships finalists	11.03 ± 0.36 11.60 ± 0.45	The mediolateral impulses decreased with reduced step width; The propulsive component of the net anteroposterior impulse is significantly smaller for the narrow step width in the 1st stance; restricting step width, vertical block impulses increased while the mediolateral motion of the CM from Start to 1st stance toe-off decreased; reducing step width does not lead to any immediate improvement in performance. On the contrary, the skating style has a greater propulsive impulse during the 1st stance	79.77
Aeles, Jonkers [9]	First stance phase. 3D kinematic full-body model. Force platform to measure the GRFs of the 1st step.	Compare young and adult sprinters in kinematic and kinetic parameters during the 1st stance phase	3D kinematics and 3D GRF of 1st step	M F ^(f)^ M F	7 9 11 10	Adult Well-trained Young Well-trained	10.67 ± 0.14 12.12 ± 0.41 11.47 ± 0.34 12.75 ± 0.36	Well-trained young and adult sprinters have no differences in ankle joint stiffness, range of dorsiflexion or plantar flexor moment. Surprisingly, the young sprinters show a greater maximal and mean ratio of horizontal to total GRF. Adult sprinters have more MTU shortening and higher maximal MTU shortening velocities in all plantar flexors and in the rectus femoris.	80.68
Brazil, Exell [11]	Block phase. Force instrumented starting blocks. Three-dimensional kinematic lower limb model.	Explore the relationships between lower limb joint kinetics, external force production and starting block performance	3D block reaction forces and 3D kinematics	M	17	Sprinters	10.67 ± 0.32	86% of the variation in block performance is explained by the horizontal force applied to the front and rear blocks, and at the joint level 55% of the variation in block performance is explained by average rear ankle extensor moment, front hip extensor moment and front knee positive extensor power.	87.73
Brazil, Exell [4]	Block phase and 1st stance. Three-dimensional kinematic lower limb model. Force platform to the GRFs—1st step.	Examine lower limb joint kinetics during the block and 1st stance phases	3D kinematics and 3D block and 1st step reaction forces	M	10	Sprinters	10.50 ± 0.27	The asymmetrical nature of the block phase is most pertinent at the knee joint, and the leg extensor energy is predominantly generated at the hip joint in both the front and the rear block whereas during 1st stance, energy generation favors the ankle joint as a result of a significant reduction in relative hip work.	83.18
Ciacci, Merni [38]	Block phase and first 2 steps. 3D kinematic full-body model.	Compare kinematic differences between sexes	3D kinematic data	M F M F	6 6 4 4	Elite World-Class	10.74 ± 0.21 11.95 ± 0.24 10.03 ± 0.14 11.10 ± 0.17	The start kinematics is only partially affected by sex (men have shorter pushing phase, higher block horizontal velocity, and shorter contact times of first 2 steps), whereas a bigger role is played by the performance level (faster sprinters have CM closer to the ground and a more flexed front knee in the “set” position, longer pushing phase, lower block vertical velocity, and shorter contact times/longer flight times in first 2 steps.	85.23
Coh, Peharec [5]	Block phase and first 2 steps. Two independent force platforms for 2 independent starting block pads. 3D kinematic full-body model.	Compare the kinematic and kinetic factors between faster and slower high-level sprinters	3D GRF under each block and spatiotemporal data	M	6 6	Faster Slower	10.66 ± 0.18 11.00 ± 0.06	Faster sprinters show motor patterns of greater force development (rear block total force, rear block vertical maximal force, and the rate of force development) than their slower counterparts; The importance of other indicators as block clearance time, block velocity, and block acceleration was not confirmed in this study.	78.41
Debaere, Vanwanseele [15]	Block phase through until the start of 2nd touchdown. 3D kinematic full-body model and 2 force platforms for the first 2 steps.	Compare joint power generation between well-trained adult and young sprinters	3D Kinematics and 3D GRF of the first 2 steps	M F M F M F	8 6 8 10 5 6	Well-Trained Adult Under 18 Under 16	10.65 ± 0.07 11.87 ± 0.14 11.21 ± 0.11 12.42 ± 0.25 11.56 ± 0.08 12.86 ± 0.30	Adult sprinters generated more joint power at the knee during the 1st step compared to young sprinters, inducing longer step length and therefore higher velocity. Younger athletes employed a different technique: the hip contributes more to total power generation, whereas the contribution of the knee is far less.	82.95
Schrodter, Bruggemann [25]	Block phase. 2D ankle kinematic data and 3D block reaction force from instrumented blocks.	Describe the stretch-shortening behavior of ankle plantarflexion MTU during the push-off phase	2D kinematics and 3D block GRF	M ^(e)^ F	54 30	World-class National	10.98 ± 0.58 12.12 ± 0.68	This study provided the 1st systematic observation of ankle joint stretch-shortening behavior during sprint start for sprinters of a wide range of performance levels. It showed clear signs of a dorsi-flexion in the front and rear ankle joints preceding plantarflexion, seeming that the stretch-shortening cycle like the motion of the soleus muscle-tendon unit has an enhancing influence on push-off force generation.	68.86
Chen, Wu [37]	Block phase. Two-dimensional kinematic full-body model (15 segmented model).	Identifies optimal crouched position (bunched, medium, or elongated) from push-off through the first 2 steps	2D kinematic data—sagittal plane and spatiotemporal data	M	7	Skilled sprinters	10.94 ± 0.20	The medium starting position was the ideal starting position.	60.19
Bezodis, Salo [3]	Block phase. Two-dimensional kinematic full-body model and kinetic data calculated from consequent data procedures.	Identify the key characteristics of the lower-limb kinematic patterns during the block phase	2D kinematic data—(kinetic energy calculated from CM horizontal velocity)	M	16	World-class to university level	10.95 ± 0.51	Describes the lower limb joint kinematics patterns explicative of high levels of sprint start performance. The rear hip angle at block exit was highly related to block power, and there were moderate positive relationships between block power and rear hip ROM and peak angular velocity.	80.68
Debaere, Delecluse [14]	Block phase and first 2 steps. Three-dimensional kinematic full-body model and 2 force platforms for first 2 steps for inverse dynamics analysis.	Analyze the contribution of joint moments and muscle forces to the CM acceleration	3D kinematics and two 1st steps GRF	M ^(e)^ F	2 5	Well-Trained	11.10 to 11.77 12.05 to 12.36	Relates the specific joint and muscle contribution to the horizontal acceleration (propulsion) and vertical acceleration (body lift) of the CM during the initial two steps after block clearance. Torque-driven simulations identify the ankle joint as the major contributor to propulsion and body lift.	68.41
Otsuka, Kurihara [21]	Block phase. Separated starting blocks secured onto separate force platforms. Three-dimensional kinematic 7-segment model of the lower limb.	Clarify the effect of widened stance width at the “set” position during the block start phase	3D kinematic data and 2D GRF under each block	M	14	3 international 11 national	10.99 ± 0.40	A widened stance width at the “set” position affects the hip-joint kinematics in both legs and enhanced the hip power generation in the rear leg during the block start phase. However, when considering sprinting performance during the whole block start phase, there was no significant effect of the widened stance width on block-induced power and the subsequent sprint time.	68.86
Rabita, Dorel [22]	Initial sprint acceleration. Six individual force platforms connected in series.	Describe the sprint acceleration mechanics in elite and sub-elite sprinters	3D GRF of initial steps and spatiotemporal data	M	4 5	Elite Sub-Elite	9.95 to 10.29 10.40 to 10.60	Describes for the 1st time the mechanical characteristics of the acceleration phase in elite and sub-elite sprinters: (i) while step length increases regularly during the acceleration phase, step frequency is almost instantaneously leveled at the maximal possibility of elite athletes; (ii) F-V and P-V relationships during sprints were well described by linear and quadratic models, respectively; and (iii) the effectiveness of force application greatly accounts for the differences in performance among highly trained athletes.	74.31
Milanese, Bertucco [41]	Block phase and first 2 stance phases. 3D kinematic full-body model.	Investigate the rear knee angle associated with the impulse and the horizontal velocity in the starting block and acceleration phases	3D kinematics	M ^(e)^ F	6 5	University sprinters	12.0 ± 0.1 13.1 ± 0.9	Horizontal CM velocity increased significantly at the block clearance and along the first 2 strides when witching from 135° to 115° and then to 90° the rear knee angle. The horizontal velocity was directly determined by force impulse which tended to be greater at 90° rear knee angle.	79.55
Otsuka, Shim [42]	Block phase and first 2 steps. Ten individual force platforms connected in series.	Compare 3D force application in the blocks between 3 sprinting groups	3D GRF under each block and first 2 steps	M	9 9 11	Well-Trained Trained Non-Trained	10.87 ± 0.41 11.31 ± 0.42 --------	The greater anterior acceleration of well-trained sprinters during the starting block phase may be accompanied, not by a greater GRF magnitude, but by a more forward-leaning sagittal GRF vector.	72.50
Debaere, Delecluse [6]	Block phase and first 2 steps. Three-dimensional kinema-tic full-body model. Two force platforms for first 2 steps.	Characterize the sprint technique during the transition from start block into sprint running	3D kinematics and 3D GRF of the first 2 steps	M ^(e)^ F	11 10	Elite/Well-Trained	10.62 + 0.18 11.89 + 0.30	During the 1st step, maximal power was predominately generated by the hip (54%) followed by the knee (31%) and the ankle (15%). The importance of power generation at the knee decreased at second stance since it only accounted for 9% of total power generation and the importance of the ankle increased up to 38%.	64.77
Aerenhouts, Delecluse [1]	Block phase and initial acceleration (first 5 steps). Instrumented start blocks and a universal laser velocity sensor.	Compare starting performance between adults and juniors sprinters having reached their adult height	Horizontal block forces and spatiotemporal data	M F ^(g)^ M F	16 9 23 19	Elite Adult Elite Junior	10.81 ± 0.40 11.29 ± 0.29 11.85 ± 0.24 12.54 ± 0.26	The higher muscularity of senior athletes did not result in significantly higher forces against the starting blocks nor block velocity compared with the junior athletes. The more muscular senior athletes had a better running acceleration than the junior athletes. In female athletes, a higher body fat percentage negatively correlated with 1st step length.	79.32
Slawinski, Dumas [8]	Block phase and 1st step. Three-dimensional kinematic full-body model.	Compare the influence of bunched, medium, and elongated start on start performance	3D kinematics and spatiotemporal data	M ^(e)^ F	6 3	National sprinters	10.58 ± 0.27 11.61 ± 0.42	Head and trunk limb movements were important to create a high CM velocity during the starting block phase. The elongated start, compared to the bunched or medium start, induced an increase in block velocity and a decrease in the time at 5 and 10 m.	72.73
Bezodis, Salo [2]	Block Phase. High-speed camera and a laser distance measurement device.	Choose the measure that best describes sprint start performance	Spatiotemporal data and horizontal block forces derivations	M	12	University sprinters	11.30 ± 0.42	For the 1st time, normalized average horizontal external power was identified as the most appropriate measure of performance. One single measure reflects how much a sprinter is able to increase velocity and the time taken to achieve this, whilst accounting for variations in morphologies between sprinters.	79.55
Slawinski, Bonnefoy [7]	Block phase and first 2 steps. Three-dimensional kinematic full-body model.	Identify the most relevant kinematic and kinetic parameters differentiators of elite and well-trained sprinters	3D kinematics and spatiotemporal data	M	6 6	Elite Well-Trained	10.27 ± 0.14 11.31 ± 0.28	Anterior and vertical components of CM, rate of force development and force impulse were significantly greater in elite sprinters. The muscular strength and arm coordination appear to characterize the efficiency of the sprint start.	67.73
Slawinski, Bonnefoy [36]	Block phase. Three-dimensional kinematic full-body model.	Measure the joint angular velocity and the kinetic energy of the different segments in elite sprinters	3D kinematics and 3D Euler angular velocities	M	8	Elite	10.30 ± 0.14	Highlights the importance of a 3D analysis of a sprint start. Joints such as shoulders, thoracic, or hips did not reach their maximal angular velocity with a movement of flexion-extension, but with a combination of flexion–extension, abduction–adduction and internal–external rotation.	67.73
Maulder, Bradshaw [40]	Block phase and first 3 steps. Two-dimensional kinematic full-body model.	Examine the changes in block start and early sprint acceleration kinematics with resisted sled loading	2D kinematics—Sagittal plane	M	10	National and Regional	10.87 ± 0.36	A load of approximately 10% BM had no ‘‘negative’’ effect on sprint start technique or step kinematic variables. The kinematic changes produced by the 10% BM load may be more beneficial than those of the 20% BM load.	76.14
Gutierrez-Davilla, Dapena [17]	Block phase. Two synchronized force platforms under blocks (1) and hands (2)	Compare the CM velocities and positions between pre-tensed and conventional starts	Horizontal forces and spatiotemporal data	M	19	Experienced competitive sprinters	11.09 ± 0.30	The pre-tensed and conventional starts produced similar performance. The increased propulsive force exerted through the legs in the early part of the block acceleration phase in the pre-tensed starts was counteracted by an increased backward force exerted through the hands during the same period.	72.95
Mero, Kuitunen [19]	“Set” position (block phase and 1st step). Sixteen individual force platforms connected in series.	Examine the effects of muscle-tendon length on joint moment and power	2D kinematics and horizontal and vertical GRF under blocks, hands and 1st step	M	9	Sprinters	10.86 + 0.34	Lower block angles (40° vs. 65°) were associated with enhanced starting performance by increasing the final block velocity. The inverse association between block angles and muscle-tendon lengths of the gastrocnemius and soleus in both legs, which may generate higher peak joint moments and powers, especially in the ankle joint, may explain this result.	68.86
Fortier, Basset [16]	Block phase and first 2 steps. Three-dimensional full-body kinematic model. Instrumented blocks.	Examine if kinetic and kinematic parameters could differentiate elite from sub-elite sprinters	3D kinematics and horizontal block forces	M M	6 6	Elite Sub-Elite	<10.70 10.70 to 11.40	Four kinetic parameters differentiating elite from sub-elite sprinters: delay between the end of rear and front force offset, rear peak force, total block time, and time to rear peak force.	72.73
Čoh, Jost [13]	Block phase and first 2 steps. Two-dimensional kinematic full-body model. Instrumented blocks.	Determine the most important kinematic and kinetic parameters of the “set” position and push-off	Horizontal block forces, 2D kinematic and spatiotemporal data	M F	13 11	Slovene national team	10.73 ± 0.2 11.97 ± 2.6	Identification of three parameters that best define an efficient start for both male and female sprinters: horizontal start velocity, start reaction time and impulse of push-off force from the front starting block.	65.22
Guissard, Duchateau [34]	Block phase. Strain gauges mounted on each footplate and behind the starting block. Two-dimensional kinematic front leg model.	Analyze the mechanical parameters about EMG activity at different front block inclinations	EMG, 2D kinematics and horizontal GRF behind blocks	M F	14 3	Trained	10.4 to 11.9	Decreasing front block obliquity induced neural and mechanical modifications that contribute to increasing the block start velocity without any increase in the duration of the push-off phase.	76.36
Mero [18]	Block phase and 1st stance. Starting blocks over a force platform. Two-dimensional kinematic full-body model (14 points).	Analyze the force-time characteristics during the 1st stance and the relationships between force and run velocity	2D kinematics and horizontal and vertical GRF under blocks and 1st step	M	8	Trained	10.79 ± 0.21	In the 1st contact after leaving the blocks there was a significant braking phase and the force produced in the propulsion phase was associated with running velocity; Muscle strength strongly affected running velocity in sprint start.	57.27

2D—two-dimensional analysis; 3D—three-dimensional analysis; AHEP—average horizontal external power; BM—body mass; CM—center of mass; COP—center of pressure; EMG—electromyography; F—female sample; F-V—force-velocity; GRF—ground reaction forces; MTU—muscle-tendon unit; M—male sample; P-V—power-velocity; ROM—range of motion; WR—world record; ^(a)^ 100 m world record at the study time was 9.58 s; ^(b)^ 100 m U20 world record at the study time was 9.97 s; ^(c)^ all sample was divided into 3 groups according to the Cormic Index (12 brachycormic, 19 metricormic, and 11 macrocormic); ^(d)^ sample divided into two groups: 5 elite sprinters and remaining 52 sprinters; ^(e)^ all subjects included in a single experimental group; ^(f)^ sample divided into 2 experimental groups: adult/senior vs. junior sprinters; ^(g)^ sample divided into 4 experimental groups.

## Data Availability

Not applicable.

## Appendix A

**Table A1:** Summary of the kinematic variables in the “Set” position. Data are the magnitude of the mean ± SD presented in the reviewed studies. Groups are male, female, and mixed (when authors joined data without discriminating by sex) sprinters. Studies are listed, in each variable, in reverse chronological order. Data, terms, conditions, and sprinters’ performance levels are presented according to the original authors. Statistical differences between groups are marked with asterisks (* *p* < 0.05; *** *p* < 0.001).

“Set” Position Kinematics	Study	Male						Female		Mixed					
Inter-block spacing (cm) (m)	Cavedon, Sandri [12]									Usual condition 27.6 ± 2.4 cm			Anthropometric condition 36.8 ± 2.3 cm		
	Čoh, Jost [13]	Slovene national sprinters 26.72 ± 2.33 cm						Slovene national sprinters 23.47 ± 1.88 cm ***							
	Mero [18]	Trained sprinters 0.32 ± 0.05 m													
Front block distance (to start line) (cm) (m)	Nagahara, Gleadhill [35]	National level sprinters 0.439 ± 0.045 m													
	Cavedon, Sandri [12]									Usual condition 52.3 ± 4.8 cm			Anthropometric condition 49.1 ± 3.0 cm		
	Coh, Peharec [5]	Faster sprinters 0.54 ± 0.05 m			Slower sprinters 0.51 ± 0.04 m										
	Čoh, Jost [13]	Slovene national sprinters 55.15 ± 6.22 cm						Slovene national sprinters 45.49 ± 5.37 cm ***							
	Mero [18]	Trained sprinters 0.51 ± 0.05 m													
Rear block distance (to start line) (cm) (m)	Nagahara, Gleadhill [35]	National level sprinters 0.686 ± 0.049 m													
	Coh, Peharec [5]	Faster sprinters 0.84 ± 0.09 m			Slower sprinters 0.79 ± 0.07 m										
	Čoh, Jost [13]	Slovene national sprinters 81.88 ± 7.47 cm						Slovene national sprinters 68.96 ± 5.91 cm ***							
	Mero [18]	Trained sprinters 0.83 ± 0.07 m													
Horizontal projection of the CM to the starting line (cm) (m)	Ciacci, Merni [38]	World-class 0.199 ± 0.054 m			Elite 0.202 ± 0.066 m			World-class 0.162 ± 0.037 m	Elite 0.248 ± 0.056 m						
		Independent of category 0.201 ± 0.058 m						Independent of category 0.214 ± 0.066 m							
	Slawinski, Dumas [8]									Bunched start 21.7 ± 2.0 cm		Medium start 25.2 ± 1.9 cm		Elongated start 30.9 ± 3.0 cm	
	Slawinski, Bonnefoy [7]	Elite 22.9 ± 1.5 cm			Well-trained 27.8 ± 2.8 cm *										
	Gutierrez-Davilla, Dapena [17]	Normal start 0.310 ± 0.057 m			Pre-tensed start 0.346 ± 0.068 m ***										
	Čoh, Jost [13]	Slovene national sprinters 18.77 ± 5.07 cm						Slovene national sprinters 15.03 ± 3.00 cm							
	Mero [18]	Trained sprinters 0.29 ± 0.05 m													
Vertical height of CM (cm) (m)	Ciacci, Merni [38]	World-class 0.643 ± 0.025 m			Elite 0.655 ± 0.038 m			World-class 0.533 ± 0.032 m	Elite 0.587 ± 0.037 m						
		Independent of category 0.650 ± 0.033 m						Independent of category 0.565 ± 0.044 m *							
	Chen, Wu [37]	Bunched start 0.57 ± 0.04 m		Medium start 0.56 ± 0.03 m		Elongated start 0.57 ± 0.03 m									
	Slawinski, Dumas [8]									Bunched start 66.6 ± 2.4 cm		Medium start 66.5 ± 2.9 cm		Elongated start 65.5 ± 2.9 cm	
	Slawinski, Bonnefoy [7]	Elite 65.7 ± 3.8 cm			Well-trained 62.6 ± 3.9 cm										
	Čoh, Jost [13]	Slovene national sprinters 54.38 ± 4.81 cm						Slovene national sprinters 53.18 ± 2.04 cm							
	Mero [18]	Trained sprinters 0.57 ± 0.04 m													
Front leg hip angle (°)	Cavedon, Sandri [12]									Usual condition 47 ± 6 ^(a)^			Anthropometric condition 43 ± 6 ^(a)^		
	Ciacci, Merni [38]	World-class 37.6 ± 0.6 ^(a)^			Elite 44.9 ± 3.3 ^(a)^			World-class 48.4 ± 14.6 ^(a)^	Elite 46.7 ± 7.5 ^(a)^						
		Independent of category 42.0 ± 4.5 ^(a)^						Independent of category 47.4 ± 10.1 ^(a)^							
	Bezodis, Salo [3]	World-class to university sprinters 47 ± 6 ^(a)^													
	Debaere, Delecluse [6]									Elite sprinters 82.8 ± 10.1 ^(b)^					
	Mero, Kuitunen [19]	Block angle 40° 52 ± 2 ^(a)^			Block angle 60° 49 ± 2 ^(a)^										
	Čoh, Jost [13]	Slovene national sprinters 44.78 ± 6.15 ^(a)^						Slovene national sprinters 42.36 ± 9.43 ^(a)^							
	Mero [18]	Trained sprinters 39 ± 7 ^(a)^													
Front leg knee angle (°)	Cavedon, Sandri [12]									Usual condition 92 ± 9 ^(c)^			Anthropometric condition 98 ± 8 ^(c)^		
	Ciacci, Merni [38]	World-class 91.0 ± 9.8 ^(c)^			Elite 99.3 ± 10.8 ^(c)^			World-class 91.0 ± 10.1 ^(c)^	Elite 100.1 ± 9.0 ^(c)^						
		Independent of category 95.9 ± 10.7 ^(c)^						Independent of category 96.4 ± 10.0 ^(c)^							
	Bezodis, Salo [3]	World-class to university sprinters 86 ± 5 ^(c)^													
	Debaere, Delecluse [6]									Elite sprinters 94.5 ± 11.2 ^(c)^					
	Slawinski, Bonnefoy [7]	Elite 110.7 ± 9.3 ^(c)^			Well-trained 106.1 ± 13.7 ^(c)^										
	Mero, Kuitunen [19]	Block angle 40° 103 ± 2 ^(c)^			Block angle 60° 97 ± 2 ^(c)^										
	Čoh, Jost [13]	Slovene national sprinters 93.75 ± 8.26 ^(c)^						Slovene national sprinters 103.38 ± 6.97 ^(c)^ *							
	Mero [18]	Trained sprinters 96 ± 12 ^(c)^													
Front leg ankle angle (°)	Cavedon, Sandri [12]									Usual condition 92 ± 6 ^(d)^			Anthropometric condition 93 ± 7 ^(d)^		
	Bezodis, Salo [3]	World-class to university sprinters 107 ± 2 ^(d)^													
	Debaere, Delecluse [6]									Elite sprinters 82.3 ± 9.5 ^(d)^					
	Mero, Kuitunen [19]	Block angle 40° 96 ± 2 ^(d)^			Block angle 60° 111 ± 2 ^(d)^										
	Čoh, Jost [13]	Slovene national sprinters 97.55 ± 10.55 ^(d)^						Slovene national sprinters 102.65 ± 6.58 ^(d)^							
	Mero [18]	Trained sprinters 94 ± 4 ^(d)^													
Rear leg hip angle (°)	Cavedon, Sandri [12]									Usual condition 77 ± 8 ^(a)^			Anthropometric condition 84 ± 8 ^(a)^		
	Ciacci, Merni [38]	World-class 71.2 ± 5.6 ^(a)^			Elite 62.6 ± 3.7 ^(a)^			World-class 75.2 ± 14.2 ^(a)^	Elite 69.5 ± 5.1 ^(a)^						
		Independent of category 66.0 ± 6.2 ^(a)^						Independent of category 71.8 ± 9.5 ^(a)^							
	Bezodis, Salo [3]	World-class to university sprinters 77 ± 9 ^(a)^													
	Debaere, Delecluse [6]									Elite sprinters 107.1 ± 9 ^(b)^					
	Mero, Kuitunen [19]	Block angle 40° 83 ± 2 ^(a)^			Block angle 60° 79 ± 2 ^(a)^										
	Čoh, Jost [13]	Slovene national sprinters 24.91 ± 4.27 ^(e)^						Slovene national sprinters 19.25 ± 9.30 ^(e)^							
	Mero [18]	Trained sprinters 77 ± 9 ^(a)^													
Rear leg knee angle (°)	Cavedon, Sandri [12]									Usual condition 112 ± 11 ^(c)^			Anthropometric condition 117 ± 11 ^(c)^		
	Ciacci, Merni [38]	World-class 120.7 ± 9.7 ^(c)^			Elite 116.1 ± 7.6 ^(c)^			World-class 113.6 ± 20.9 ^(c)^	Elite 118.4 ± 6.6 ^(c)^						
		Independent of category 118.0 ± 8.3 ^(c)^						Independent of category 116.5 ± 13.3 ^(c)^							
	Bezodis, Salo [3]	World-class to university sprinters 109 ± 9 ^(c)^													
	Debaere, Delecluse [6]									Elite sprinters 112.8 ± 15.1 ^(c)^					
	Slawinski, Bonnefoy [7]	Elite 135.5 ± 11.4 ^(c)^			Well-trained 117.3 ± 10.1 ^(c)^ *										
	Mero, Kuitunen [19]	Block angle 40° 131 ± 2 ^(c)^			Block angle 60° 122 ± 2 ^(c)^										
	Čoh, Jost [13]	Slovene national sprinters 112.72 ± 13.31 ^(c)^						Slovene national sprinters 115.59 ± 13.86 ^(c)^							
	Mero [18]	Trained sprinters 126 ± 16 ^(c)^													
Rear leg ankle angle (°)	Cavedon, Sandri [12]									Usual condition 87 ± 6 ^(d)^			Anthropometric condition 85 ± 7 ^(d)^		
	Bezodis, Salo [3]	World-class to university sprinters 111 ± 12 ^(d)^													
	Debaere, Delecluse [6]									Elite sprinters 82.5 ± 7.8 ^(d)^					
	Mero, Kuitunen [19]	Block angle 40° 95 ± 3 ^(d)^			Block angle 60° 109 ± 3 ^(d)^										
	Čoh, Jost [13]	Slovene national sprinters 97.45 ± 10.28 ^(d)^			Slovene national sprinters 99.80 ± 6.44 ^(d)^										
	Mero [18]	Trained sprinters 96 ± 8 ^(d)^													
Trunk angle (°)	Chen, Wu [37]	Bunched start −20.4 ± 7.3 *		Medium start −14.9 ± 6.7 *		Elongated start −8.8 ± 10.8 ^(f)^ *									

CM—center of mass; ^(a)^ internal angle between the thigh and trunk in flexion/extension plane; ^(b)^ relative angle between the pelvis and the thigh according to the Biomechanical Convention [53]; ^(c)^ relative angle between the thigh and the shank according to the Medical Convention [53]; ^(d)^ relative angle between the shank and the foot according to the Biomechanical Convention [53]; ^(e)^ rear leg hip angle measured as front-rear leg angle; ^(f)^ relative angle between the vector from hip to shoulder and the horizontal plane.

## Appendix B

**Table A2:** Summary of the kinematic variables in the “Block Phase”. Data are the magnitude of the mean ± SD presented in the reviewed studies. Groups are male, female, and mixed (when authors joined data without discriminating by sex) sprinters. Studies are listed, in each variable, in reverse-chronological order, followed by alphabetically for studies published in the same year. Data, terms, conditions, and sprinters’ performance levels are presented according to the original authors. Statistical differences between groups are marked with asterisks (* *p* < 0.05; ** *p*< 0.01; *** *p* < 0.001; # Cohen’s d—large effect size (>0.8); ^§^ small effect size [0.2–0.6] of 90% confidence intervals; ^§§^ moderate effect size [0.6–1.2] of 90% confidence intervals); ^ƥ^ clearly associated with average horizontal power produced across the block phase—*p* < 0.05; ^¥^ significantly greater compared to the bunched start.

Block Phase Kinematics	Study	Male						Female		Mixed					
Block time (ms) (s)	Graham-Smith, Colyer [39]	Seniors 365 ± 18 ms			Juniors 412 ± 49 ms										
	Nagahara, Gleadhill [35]	National-level sprinters 0.369 s ^(a)^													
	Sado, Yoshioka [23]	University-level sprinters 0.36 ± 0.03 s													
	Bezodis, Walton [10]	Sprint start-trained athletes 0.391 ± 0.038 s													
	Cavedon, Sandri [12]									Usual condition 0.421 ± 0.047 s			Anthropometric condition 0.427 ± 0.038 s		
	Colyer, Graham-Smith [33]	Elite 0.360 ± 0.010 s ^(^^ƥ)^			All sample 0.390 ± 0.039 s										
	Sandamas, Gutierrez-Farewik [24]									Skating condition 0.37 ± 0.03 s			Narrow condition 0.38 ± 0.03 s		
	Brazil, Exell [4]	Athletic sprinters 0.359 ± 0.014 s													
	Ciacci, Merni [38]	World-class 0.356 ± 0.011 s			Elite 0.323 ± 0.024 s			World-class 0.356 ± 0.018 s	Elite 0.323 ± 0.024 s						
		Independent of category 0.336 ± 0.025 s						Independent of category 0.336 ± 0.027 s							
	Coh, Peharec [5]	Faster sprinters 332 ± 28.73 ms			Slower sprinters 305 ± 24.35 ms										
	Bezodis, Salo [3]	World-class to university sprinters 0.358 ± 0.022 s													
	Otsuka, Kurihara [21]	Normal condition 0.334 ± 0.031 s			Widened condition 0.330 ± 0.025 s										
	Rabita, Dorel [22]	Elite 376 ± 24 ms			Sub-elite 394 ± 13 ms #										
	Milanese, Bertucco [41]									Rear knee angle @ 90° 0.354 ± 0.015 s		@ 115° 0.348 ± 0.016 s		@ 135° 0.355 ± 0.014 s	
	Otsuka, Shim [42]	Well-trained 0.349 ± 0.019 s			Trained 0.379 ± 0.022 s *										
	Aerenhouts, Delecluse [1]	Elite Seniors 357 ± 29 ms			Elite Juniors 367 ± 28 ms			Elite Seniors 380 ± 16 ms	Elite Juniors 383 ± 19 ms						
	Slawinski, Dumas [8]									Bunched start 0.371 ± 0.016 s		Medium start 0.377 ± 0.017 s		Elongated start 0.427 ± 0.056 s	
	Slawinski, Bonnefoy [7]	Elite 0.352 ± 0.018 s			Well-trained 0.351 ± 0.020 s										
	Maulder, Bradshaw [40]	National and regional level sprinters 0.31 s ^(a)^													
	Gutierrez-Davilla, Dapena [17]	Conventional start 0.375 ± 0.028 s			Pre-tensed start 0.386 ± 0.036 s *										
	Mero, Kuitunen [19]	Block angle 40° 0.343 ± 0.036 s			Block angle 65° 0.333 ± 0.027 s										
	Fortier, Basset [16]	Elite 399 ± 21 ms			Sub-elite 422 ± 33 ms *										
	Čoh, Jost [13]	Slovene national sprinters 0.30 ± 0.03 s						Slovene national sprinters 0.34 ± 0.02 s							
	Guissard, Duchateau [34]									Block angle 30° 0.321 ± 0.023 s		Block angle 50° 0.325 ± 0.035 s		Block angle 70° 0.317 ± 0.039 s	
	Mero [18]	Trained sprinters 0.342 ± 0.022 s													
Rear leg block time (ms) (s)	Nagahara, Gleadhill [35]	National-level sprinters 0.212 ± 0.029 s													
	Sado, Yoshioka [23]	University-level sprinters 0.18 ± 0.02 s													
	Cavedon, Sandri [12]									Usual condition 0.211 ± 0.041 s			Anthropometric condition 0.212 ± 0.041 s		
	Brazil, Exell [4]	Athletic sprinters 0.193 ± 0.012 s													
	Coh, Peharec [5]	Faster sprinters 162 ± 9.47 ms			Slower sprinters 149 ± 12.40 ms *										
	Otsuka, Kurihara [21]	Normal Condition 0.175 ± 0.034 s			Widened condition 0.180 ± 0.023 s										
	Milanese, Bertucco [41]									Rear knee angle @ 90° 0.12 ± 0.01 s		@115° 0.11 ± 0.01 s		@ 135° 0.09 ± 0.02 s	
	Otsuka, Shim [42]	Well-trained 0.188 ± 0.022 s			Trained 0.187 ± 0.029 s										
	Slawinski, Bonnefoy [7]	Elite 0.154 ± 0.017 s			Well-trained 0.140 ± 0.026 s										
	Mero, Kuitunen [19]	Block angle 40° 0.188 ± 0.008 s			Block angle 65° 0.172 ± 0.015 s										
	Fortier, Basset [16]	Elite 370 ± 18 ms ^(b)^			Sub-elite 268 ± 58 ms										
	Čoh, Jost [13]	Slovene national sprinters 0.20 ± 0.02 s						Slovene national sprinters 0.18 ± 0.03 s							
Ratio rear leg time/block time (%)	Sado, Yoshioka [23]	University-level sprinters 49.7 ± 5.1													
	Bezodis, Salo [3]	World-class to university sprinters 53 ± 5													
	Milanese, Bertucco [41]									Rear knee angle @ 90° 34.62 ± 3.60		@115° 31.30 ± 3.52		@135° 28.65 ± 3.57	
	Slawinski, Bonnefoy [7]	Elite 43.5 ± 3.8			Well-trained 39.8 ± 8.1										
Block resultant velocity (m·s^−1^)	Chen, Wu [37]	Bunched start 3.32 ± 0.14		Medium start 3.36 ± 0.15		Elongated start 3.45 ± 0.22									
	Slawinski, Dumas [8]									Bunched start 2.76 ± 0.11		Medium start 2.84 ± 0.14 ^¥^		Elongated start 2.89 ± 0.13 ^¥^	
	Slawinski, Bonnefoy [7]	Elite 3.48 ± 0.05			Well-trained 3.24 ± 0.18 *										
	Fortier, Basset [16]	Elite 3.28 ± 0.19			Sub-elite 3.12 ± 0.30										
	Čoh, Jost [13]	Slovene national sprinters 3.37 ± 0.35						Slovene national sprinters 3.09 ± 0.21 *							
	Mero [18]	Trained sprinters 3.46 ± 0.32													
Block horizontal velocity (m·s^−1^)	Graham-Smith, Colyer [39]	Seniors 3.36 ± 0.15			Juniors 3.16 ± 0.18 ^§§^										
	Sado, Yoshioka [23]	University-level sprinters 3.31 ± 0.13													
	Bezodis, Walton [10]	Sprint start-trained athletes 3.12 ± 0.21													
	Cavedon, Sandri [12]									Usual condition 3.36 ± 0.35			Anthropometric condition 3.50 ± 0.39		
	Colyer, Graham-Smith [33]	Elite 3.36 ± 0.13 (ƥ)			All sample 3.30 ± 0.20										
	Ciacci, Merni [38]	World-class 4.16 ± 0.39			Elite 4.08 ± 0.08			World-class 3.11 ± 0.39	Elite 3.48 ± 0.23						
		Independent of category 4.11 ± 0.24						Independent of category 3.33 ± 0.34 *							
	Coh, Peharec [5]	Faster sprinters 3.38 ± 0.10			Slower sprinters 3.19 ± 0.19 *										
	Rabita, Dorel [22]	Elite 3.61 ± 0.08			Sub-elite 3.17 ± 0.19 #										
	Milanese, Bertucco [41]									Rear knee angle @ 90° 2.67 ± 0.26		@ 115° 2.62 ± 0.23		@ 135° 2.56 ± 0.24	
	Debaere, Delecluse [6]									Elite Sprinters 3.10 ± 0.25					
	Aerenhouts, Delecluse [1]	Elite Seniors 2.9 ± 0.3			Elite Juniors 2.9 ± 0.3			Elite Seniors 2.8 ± 0.2	Elite Juniors 2.7 ± 0.3						
	Bezodis, Salo [2]	University-level sprinters 3.28 ± 0.24													
	Maulder, Bradshaw [40]	National and regional level sprinters 3.40 ± 0.20													
	Gutierrez-Davilla, Dapena [17]	Conventional start 3.21 ± 0.22			Pre-tensed start 3.22 ± 0.24										
	Mero, Kuitunen [19]	Block angle 40° 3.39 ± 0.23			Block angle 65° 3.30 ± 0.21 **										
	Čoh, Jost [13]	Slovene national sprinters 3.20 ± 0.19						Slovene national sprinters 2.99 ± 0.23 *							
	Guissard, Duchateau [34]									Block angle 30° 2.94 ± 0.20		Block angle 50° 2.80 ± 0.23		Block angle 70° 2.37 ± 0.31	
Block vertical velocity (m·s^−1^)	Graham-Smith, Colyer [39]	Seniors 0.60 ± 0.12			Juniors 0.61 ± 0.13										
	Sado, Yoshioka [23]	University-level sprinters 0.58 ± 0.08													
	Colyer, Graham-Smith [33]	Elite 0.58 ± 0.06			All sample 0.60 ± 0.11										
	Ciacci, Merni [38]	World-class −0.21 ± 0.27 ^(c)^			Elite 0.59 ± 0.32			World-class 0.38 ± 0.06	Elite 0.52 ± 0.30						
		Independent of category 0.27 ± 0.50						Independent of category 0.47 ± 0.24							
	Chen, Wu [37]	Bunched start 0.49 ± 0.19		Medium start 0.40 ± 0.15		Elongated start 0.42 ± 0.33									
	Debaere, Delecluse [6]									Elite sprinters 0.84 ± 0.13					
	Slawinski, Bonnefoy [7]	Elite 0.52 ± 0.06			Well-trained 0.51 ± 0.14										
	Čoh, Jost [13]	Slovene national sprinters 0.69 ± 0.21						Slovene national sprinters 0.76 ± 0.19							
Block acceleration (m·s^−2^)	Coh, Peharec [5]	Faster sprinters 7.47 ± 1.34			Slower sprinters 7.35 ± 0.90										
	Otsuka, Kurihara [21]	Normal condition 9.65 ± 0.72			Widened condition 9.73 ± 0.59										
	Otsuka, Shim [42]	Well-trained 9.72 ± 0.36			Trained 8.41 ± 0.49 *										
	Aerenhouts, Delecluse [1]	Elite Seniors 8.2 ± 0.9			Elite Juniors 7.9 ±0.7			Elite Seniors 7.3 ± 0.7	Elite Juniors 7.0 ± 0.8						
	Bezodis, Salo [2]	University-level sprinters 9.14 ± 0.99													
	Slawinski, Bonnefoy [7]	Elite 9.5 ± 0.4			Well-trained 8.8 ± 0.8										
	Maulder, Bradshaw [40]	National and regional level sprinters 8.00 ± 0.80													
	Guissard, Duchateau [34]									Block angle 30° 9.03 ± 0.91		Block angle 50° 8.36 ± 1.17		Block angle 70° 7.46 ± 1.42	
Take-off angle (°) ^(d)^	Milanese, Bertucco [41]									Rear knee angle @ 90° 40.42 ± 2.74		@ 115° 40.23 ± 2.13		@ 135° 39.77 ± 2.50	
	Slawinski, Bonnefoy [7]	Elite 34.7 ± 1.4			Well-trained 34.3 ± 2.0										
	Maulder, Bradshaw [40]	National and regional level sprinters 42 ± 4													
	Čoh, Jost [13]	Slovene national sprinters 49.54 ± 2.91						Slovene national sprinters 53.20 ± 3.20 *							
CM projection angle (°) ^(e)^	Graham-Smith, Colyer [39]	Seniors 10.2 ± 2.0			Juniors 11.0 ± 2.1 ^§^										
	Colyer, Graham-Smith [33]	Elite 9.8 ± 0.8 (h)			All sample 10.3 ± 2.0										
Horizontal CM ROM (m)	Gutierrez-Davilla, Dapena [17]	Conventional start 0.600 ± 0.046			Pre-tensed start 0.619 ± 0.059 *										
Angular displacement (°)															
Trunk	Bezodis, Salo [3]	World-class to university sprinters 46 ± 8													
Front hip		World-class to university sprinters 113 ± 9													
Front knee		World-class to university sprinters 73 ± 7													
Front ankle		World-class to university sprinters 36 ± 10													
Rear hip		World-class to university sprinters 31 ± 13 ^(f)^													
Rear knee		World-class to university sprinters 18 ± 6 ^(f)^													
Rear ankle		World-class to university sprinters 19 ± 9 ^(f)^													
Ankle joint dorsiflexion (°)	Schrodter, Bruggemann [25]	Front block ^(g)^ 15.8 ± 7.4			Rear block ^(g)^ 8.0 ± 5.7 ***										
Trunk angle at takeoff (°) ^(h)^	Chen, Wu [37]	Bunched start 25.7 ± 6.1		Medium start 29.1 ± 4.5 *		Elongated start 28.9 ± 4.5									
	Maulder, Bradshaw [40]	National and regional level sprinters 22 ± 7													
Hip angle at takeoff (°)	Debaere, Delecluse [6]									Elite sprinters—Rear block 146.8 ± 9.4 ^(i)^			Elite sprinters—Front block 183.2 ± 6.8 ^(i)^		
Knee angle at takeoff (°)										Elite sprinters—Rear block 134.9 ± 11.2 ^(j)^			Elite sprinters—Front block 177.4 ± 5.2 * ^(j)^		
Ankle angle at takeoff (°)										Elite sprinters—Rear block 139.2 ±7.0 ^(k)^			Elite sprinters—Front block 133.1 ± 6.7 ^(k)^		
Joint angular velocity (°.s^−1^)															
Trunk	Slawinski, Bonnefoy [36]	Elite sprinters 220.2 ± 57.5													
Front hip		Elite sprinters 456.3 ± 17.7													
Front knee		Elite sprinters 660.2 ± 40.5													
Front ankle		Elite sprinters 641.5 ± 44.9													
Rear hip		Elite sprinters 425.7 ± 61.0													
Rear knee		Elite sprinters 651.4 ± 112.3													
Rear ankle		Elite sprinters 462.9 ± 74.7													

CM—center of mass; ROM—range of motion; ^(a)^ block time calculated from the difference between the average data of total block time and reaction time data; ^(b)^ probably an incorrect data from the original paper; ^(c)^ presumably the negative signal is a gap in the data reported in the original paper; ^(d)^ the take-off or push-off angle is the angle between the horizontal and the line passing through the most front part of the contact foot and the center of mass at block clearance; ^(e)^ center of mass projection angle is calculated as the resultant direction from the horizontal and vertical block exit velocities of the center of mass; ^(f)^ angular displacement during rear block contact only; ^(g)^ higher magnitude of dorsiflexion was correlated to a faster stretch velocity, which was related to increased force generation (maximal rate of force development, maximal resultant and horizontal push force, and also normalized average horizontal block power); ^(h)^ the angle, measured relative to the horizontal, between the line passing through the hip and shoulder (trunk segment) of the side of the body in which the athlete’s front foot at the block take off instant; ^(i)^ relative angle between the pelvis and the thigh according to the Biomechanical Convention [53]; ^(j)^ relative angle between the thigh and the shank according to the Medical Convention [53]; ^(k)^ relative angle between the shank and the foot according to the Biomechanical Convention [53].

**Table A3:** Summary of the kinetic variables in the “Block Phase”. Data are the magnitude of the mean ± SD presented in the reviewed studies. Groups are male and mixed (when authors joined data without discriminating by sex) sprinters. Studies are listed, in each variable, in reverse-chronological order, followed by alphabetically for studies published in the same year. Data, terms, conditions, and sprinters’ performance levels are presented according to the original authors. Statistical differences between groups are marked with asterisks (* *p* < 0.05; ** *p* < 0.01; *** *p* < 0.001; # Cohen’s d—large effect size (>0.8); ^§§§^ large effect size [1.2–1.6] of 90% confidence intervals; ^ƥ^ clearly associated with average horizontal power produced across the block phase—*p* < 0.05; ^ƥƥ^ moderately associated (moderate effect size: >0.3) with average horizontal power produced across the block phase).

Block Phase Kinetics			Male						Mixed					
Block force	Initial force on blocks—“Set” position (N.N^−1^)	Gutierrez-Davilla, Dapena [17]	Normal start 0.113 ± 0.04			Pre-tensed start 0.186 ± 0.053 ***								
	Relative average total force (NAF) (N·kg^−1^)	Sandamas, Gutierrez-Farewik [24]							Skating conditions 1.44 ± 0.07 BW ^(a)^			Narrow condition 1.44 ± 0.07 BW ^(a)^		
		Cavedon, Sandri [12]							Usual condition 11.37 ± 1.19			Anthropometric condition 11.55 ± 1.12		
		Otsuka, Shim [42]	Well-trained sprinters 15.03 ± 0.32			Trained sprinters 13.99 ± 0.65								
	Average horizontal force (AHF) (N)	Rabita, Dorel [22]	Elite sprinters 783 ± 59			Sub-elite sprinters 596 ± 47 ^#^								
		Mero [18]	Trained sprinters 655 ± 76											
	Relative average horizontal force (NAHF) (N·kg^−1^) (BW)	Colyer, Graham-Smith [33]	Elite sprinters 9.4 ± 0.1 N·kg^−1 (^^ƥ)^			All sample 8.7 ± 1.1 N·kg^−1^								
		Sandamas, Gutierrez-Farewik [24]							Skating condition 0.87 ± 0.10 BW ^(a) (b)^			Narrow condition 0.86 ± 0.10 BW ^(a) (b)^		
		Rabita, Dorel [22]	Elite sprinters 9.59 ± 0.53 N·kg^−1^			Sub-elite sprinters 7.74 ± 0.82 N·kg^−1 #^								
		Otsuka, Shim [42]	Well-trained sprinters 9.72 ± 0.36 N·kg^−1^			Trained sprinters 8.41 ± 0.49 N·kg^−1^ *								
Peak-to-minimum horizontal force average change (N·kg^−1^) (transition from bilateral to unilateral pushing)		Colyer, Graham-Smith [33]	Elite sprinters −10.3 ± 3.1 ^(^^ƥ)^			All sample −10.6 ± 2.5								
Resultant force front block resultant force (N)		Coh, Peharec [5]	Faster sprinters 1104 ± 82.53			Slower sprinters 1073 ± 56.21								
Relative front block resultant mean force (N·kg^−1^)		Nagahara, Gleadhill [35]	Normal condition 9.25 ± 0.39		Anterior condition 9.03 ± 0.63		Posterior condition 9.44 ± 0.84							
		Otsuka, Shim [42]	Well-trained sprinters 10.03 ± 1.07			Trained sprinters 9.62 ± 0.94								
Rear block resultant force (N)		Coh, Peharec [5]	Faster sprinters 913 ± 89.23			Slower sprinters 771 ± 55.09 **								
Relative rear block resultant mean force (N·kg^−1^)		Nagahara, Gleadhill [35]	Normal condition 7.20 ± 0.52		Anterior condition 6.05 ± 1.55		Posterior condition 8.23 ± 1.13 * ^(c)^							
		Otsuka, Shim [42]	Well-trained sprinters 7.71 ± 1.24			Trained sprinters 7.46 ± 1.04								
Horizontal force														
Front block horizontal maximal force (N)		Coh, Peharec [5]	Faster sprinters 461 ± 51.05			Slower sprinters 398 ± 56.73								
		Aerenhouts, Delecluse [1]	Elite Seniors 686 ± 110			Elite Juniors 623 ± 105			Elite Seniors ^(d)^ 482 ± 98			Elite Juniors ^(d)^ 454 ± 65		
Relative front block horizontal maximal force (N·kg^−1^)		Cavedon, Sandri [12]							Usual condition 6.02 ± 0.71			Anthropometric condition 5.91 ± 0.65		
Relative front block horizontal mean force (N·kg^−1^)		Nagahara, Gleadhill [35]	Normal condition 5.87 ± 0.38		Anterior condition 5.93 ± 0.56		Posterior condition 6.25 ± 0.64							
		Otsuka, Shim [42]	Well-trained sprinters 6.70 ± 0.58			Trained sprinters 5.99 ± 0.67								
Rear block horizontal maximal force (N)		Coh, Peharec [5]	Faster sprinters 460 ± 58.12			Slower sprinters 423 ± 45.50								
		Aerenhouts, Delecluse [1]	Elite Seniors 785 ± 220			Elite Juniors 697 ± 143			Elite Seniors ^(d)^ 485 ± 986			Elite Juniors ^(d)^ 435 ± 115		
Relative rear block horizontal maximal force (N·kg^−1^)		Cavedon, Sandri [12]							Usual condition 4.52 ± 1.09			Anthropometric condition 4.95 ± 1.34 *		
Relative rear block horizontal mean force (N·kg^−1^)		Nagahara, Gleadhill [35]	Normal condition 5.18 ± 0.38		Anterior condition 3.97 ± 1.17		Posterior condition 6.14 ± 0.86 ** ^(c)^							
		Otsuka, Shim [42]	Well-trained sprinters 5.82 ± 0.71			Trained sprinters 5.41 ± 0.88								
Vertical force														
Front block vertical maximal force (N)		Coh, Peharec [5]	Faster sprinters 1019 ± 69.99			Slower sprinters 978 ± 43.12								
Relative front block vertical maximal force (N·kg^−1^)		Cavedon, Sandri [12]							Usual condition 6.13 ± 0.92			Anthropometric condition 6.12 ± 0.90		
Relative front block vertical mean force (N·kg^−1^)		Nagahara, Gleadhill [35]	Normal condition 7.15 ± 0.29		Anterior condition 6.81 ± 0.40		Posterior condition 7.07 ± 0.64							
		Otsuka, Shim [42]	Well-trained sprinters 7.43 ± 1.01			Trained sprinters 7.50 ± 0.78								
Rear block vertical maximal force (N)		Coh, Peharec [5]	Faster sprinters 795 ± 91.29			Slower sprinters 645 ± 41.55 **								
Relative rear block vertical maximal force (N·kg^−1^)		Cavedon, Sandri [12]							Usual condition 3.78 ± 1.12			Anthropometric condition 3.96 ± 1.20		
Relative rear block vertical mean force (N·kg^−1^)		Nagahara, Gleadhill [35]	Normal condition 4.99 ± 0.57		Anterior condition 4.53 ± 1.17		Posterior condition 5.47 ± 0.83							
		Otsuka, Shim [42]	Well-trained sprinters 5.03 ± 1.15			Trained sprinters 5.12 ± 0.68								
Maximal rate of force development (N·s^−1^) (N·kg^−1^·s^−1^)		Schrodter, Bruggemann [25]							World-class sprinters 259 ± 79 N·kg^−1^.s^−1^			Well-trained sprinters 175 ± 86 N·kg^−1^.s^−1^ **		
		Slawinski, Bonnefoy [7]	Elite 15505 ± 5397 N·s^−1^			Well-trained 8459 ± 3811 N·s^−1^ *								
Block power	Average horizontal block power (W) ^(e)^	Bezodis, Walton [10]	Sprint start-trained athletes 832 ± 113											
		Bezodis, Salo [3] ^(f)^	World-class to university sprinters 1171 ± 268											
		Rabita, Dorel [22]	Elite sprinters 1415 ± 118			Sub-elite sprinters 949 ± 124 ^#^								
		Bezodis, Salo [2] ^(f)^	University-level sprinters 1094 ± 264											
	Relative average horizontal external power (W·kg^−1^)	Graham-Smith, Colyer [39]	Seniors sprinters 15.5 ± 1.5			Juniors sprinters 12.4 ± 2.2 ^§§§^								
		Nagahara, Gleadhill [35]	Normal condition 14.8 ± 1.0		Anterior condition 13.2 ± 1.3		Posterior condition 16.2 ± 2.1 ** ^(c)^							
		Colyer, Graham-Smith [33]	Mix of elite, senior and junior sprinters 14.3 ± 2.3											
		Nagahara and Ohshima [20]	Sprinters 14.7 ± 1.4											
		Rabita, Dorel [22]	Elite sprinters 17.3 ± 1.3			Sub-elite sprinters 12.3 ± 1.9 ^#^								
	Normalized average horizontal external power ^(g)^	Sado, Yoshioka [23]	University-level sprinters 0.55 ± 0.05											
		Bezodis, Walton [10]	Sprint start-trained athletes 0.43 ± 0.06 (associated with block velocity)											
		Cavedon, Sandri [12]							Usual condition 0.47 ± 0.90			Anthropometric condition 0.50 ± 0.10 *		
		Sandamas, Gutierrez-Farewik [24]							Skating condition 0.46 ± 0.07			Narrow condition 0.45 ± 0.07		
		Schrodter, Bruggemann [25]							World-class sprinters 0.360 ± 0.098			Well-trained sprinters 0.305 ± 0.056 ** ^(h)^		
		Bezodis, Salo [3]	World-class to university sprinters 0.53 ± 0.08 (associated with PB100m)											
		Otsuka, Kurihara [21]	Normal condition 0.539 ± 0.053			Widened condition 0.543 ± 0.051								
		Bezodis, Salo [2]	University-level sprinters 0.51 ± 0.09 (associated with block velocity and acceleration data)											
Force impulse	Absolute force impulse (N·s)	Coh, Peharec [5]	Faster sprinters 294.3 ± 21.1			Slower sprinters 269.5 ± 17.9 *								
		Milanese, Bertucco [41]							Rear knee angle @ 90° 175.00 ± 26.49		@ 115° 172.00 ± 25.49		@ 135° 168.35 ± 25.61	
		Slawinski, Bonnefoy [7]	Elite sprinters 276.2 ± 36.0			Well-trained sprinters 215.4 ± 28.5 *								
		Mero, Kuitunen [19]	Block angle 40° 249.0 ± 21.5			Block angle 65° 240.3 ± 22.9								
	Relative force impulse (N·s·kg^−1^) (m·s^−1^)	Cavedon, Sandri [12]							Usual condition 4.76 ± 0.55 N·s·kg^−1^			Anthropometric condition 4.93 ± 0.56 N·s·kg^−1^		
		Sandamas, Gutierrez-Farewik [24]							Skating conditions 3.27 ± 0.15 m·s^−1^			Narrow conditions 3.25 ± 0.16 m·s^−1^		
	Horizontal force impulse (N·s)	Coh, Peharec [5]	Faster sprinters 140.7 ± 11.5			Slower sprinters 112.8 ± 10.4 ***								
		Mero [18]	Trained sprinters 223 ± 18											
	Relative horizontal force impulse (m·s^−1^) (N·s·kg^−1^)	Sandamas, Gutierrez-Farewik [24]							Skating condition 3.21 ± 0.16 m·s^−1^			Narrow condition 3.19 ± 0.16 m·s^−1^		
		Otsuka, Kurihara [21]	Normal condition 3.20 ± 0.18 N·s·kg^−1^			Widened condition 3.20 ± 0.20 N·s·kg^−1^								
		Otsuka, Shim [42]	Well-trained sprinters 3.407 ± 0.149 N·s·kg^−1^			Trained sprinters 3.179 ± 0.163 N·s·kg^−1^								
	Vertical force impulse (N·s)	Coh, Peharec [5]	Faster sprinters 256.1± 9.7			Slower sprinters 209.8 ± 8.9 ***								
		Mero [18]	Trained sprinters 173 ± 30											
	Normalized vertical force impulse (m·s^−1^)	Sandamas, Gutierrez-Farewik [24]							Skating condition 0.54 ± 0.07			Narrow condition 0.59 ± 0.08 *		
	Normalized me-diolateral force impulse (m·s^−1^)	Sandamas, Gutierrez-Farewik [24]							Skating conditions 0.23 ± 0.10			Narrow condition 0.08 ± 0.05 *		
	Force impulse of front block (N·s)	Coh, Peharec [5]	Faster sprinters 221.3 ± 15.8			Slower sprinters 178.3 ± 13.1 ***								
	Force impulse of rear block (N·s)	Coh, Peharec [5]	Faster sprinters 76.7 ± 8.8			Slower sprinters 71.1 ± 6.7								
COP location (m)	Front block anteroposterior location	Nagahara and Ohshima [20]	Sprinters −0.080 ± 0.024 ^(^^ƥƥ)^											
	Front block vertical location		Sprinters 0.061 ± 0.022 ^(^^ƥ)^											
	Rear block anteroposterior location		Sprinters −0.082 ± 0.018											
	Rear block vertical location		Sprinters 0.064 ± 0.018 ^(^^ƥ)^											
	Front block location		Sprinters −0.45 ± 0.05 ^(^^ƥƥ)^											
	Rear block location		Sprinters −0.69 ± 0.06											
Peak joint moments	Peak ankle ex-tension moment	Brazil, Exell [4]	Rear block 0.236 ± 0.044 ^(i)^			Front block 0.172 ± 0.032 ^(i)^ *								
	Peak knee exten-sion moment		Rear block 0.054 ± 0.020 ^(i)^			Front block 0.199 ± 0.067 ^(i)^ *								
	Peak hip exten-sion moment		Rear block 0.315 ± 0.086 ^(i)^			Front block 0.349 ± 0.035 ^(i)^								
	Peak lumbosa-cral extension moment (N·s^−1^)	Sado, Yoshioka [23]	University-level sprinters 3.64 ± 0.39 ^(j) (^^ƥ)^											
Peak joint powers	Peak positive ankle power	Brazil, Exell [4]	Rear block 0.236 ± 0.066 ^(i)^			Front block 0.388 ± 0.084 ^(i)^ *								
	Peak positive knee power		Rear block 0.047 ± 0.026 ^(i)^			Front block 0.440 ± 0.177 ^(i)^ *								
	Peak positive hip power		Rear block 0.408 ± 0.152 ^(i)^			Front block 0.576 ± 0.071 ^(i)^ *								

^(a)^ Normalized to body mass, gravity constant and sprinter’s leg length; ^(b)^ units as reported in the original article; ^(c)^ significantly different from the anterior condition; ^(d)^ only elite female data; ^(e)^ average horizontal external power is calculated as the product of anteroposterior force and horizontal velocity; ^(f)^ average horizontal external power was calculated based on the rate of change of mechanical energy in a horizontal direction (i.e., change in kinetic energy divided by time) [2]; ^(g)^ normalized average horizontal external power is the average horizontal external power normalized to the mass and the leg length of the sprinter [2]; ^(h)^ for normalization, the body height was used instead of the sprinter’s leg length [25]; ^(i)^ joint data normalized to the mass and the leg length of the sprinter; ^(j)^ significantly larger (*p* < 0.05, Cohen’s *d* = 2.02–11.09) than any other lower-limb and lumbosacral torques, although quantitative data for the remaining joint torques are not available.

## Appendix C

**Table A4:** Summary of the kinematic variables in the “first two steps”. Data are the magnitude of the mean ± SD presented in the reviewed studies. Groups are male, female, and mixed (when authors joined data without discriminating by sex) sprinters. Studies are listed, in each variable, in reverse-chronological order, followed by alphabetically for studies published in the same year. Data, terms, conditions, and sprinters’ performance levels are presented according to the original authors. Statistical differences between groups are marked with asterisks (* *p* < 0.05; ** *p* < 0.01; *** *p* < 0.001; ^#^ significant different from adults; ^§^ small effect size [0.2–0.6] of 90% confidence intervals; ^§§^ moderate effect size [0.6–1.2] of 90% confidence intervals).

First and Second Steps Kinematics	Study	Male						Female		Mixed					
**First Step**															
First step length (m) (cm)	Cavedon, Sandri [12]									Usual condition 1.09 ± 0.11 nor. to leg length			Anthropometric condition 1.12 ± 0.12 nor. to leg length		
	Ciacci, Merni [38]	World-class 1.135 ± 0.025 m			Elite 0.968 ± 0.162 m			World-class 1.068 ± 0.032 m	Elite 0.950 ± 0.099 m						
		Independent of category 1.035 ± 0.149 m						Independent of category 0.997 ± 0.097 m							
	Coh, Peharec [5]	Faster sprinters 1.30 ± 0.51 m			Slower sprinters 1.06 ± 0.60 m ^§^										
	Debaere, Vanwanseele [15]									Adult sprinters 1.00 ± 0.07 m		U18 sprinters 0.94 ± 0.11 m		U16 sprinters 0.94 ± 0.10 m	
	Chen, Wu [37]	Bunched start 0.97 ± 0.10 m		Medium start 1.00 ± 0.12 m *		Elongated start 1.03 ± 0.10 m *									
	Bezodis, Salo [3]	World-class to university sprinters 1.10 ± 0.07 normalized to step length													
	Rabita, Dorel [22]	Elite 0.96 ± 0.16 m			Sub-elite 1.01 ± 0.06 m										
	Milanese, Bertucco [41]									Rear knee angle @ 90° 1.23 ± 0.12 m		@ 115° 1.22 ± 0.11 m		@ 135° 1.21 ± 0.13 m	
	Aerenhouts, Delecluse [1]	Elite Seniors 85 ± 33 cm			Elite Juniors 63 ±27 cm *			Elite Seniors 82 ± 19 cm	Elite Juniors 61 ± 20 cm *						
	Slawinski, Bonnefoy [7]	Elite 137.1 ± 9.0 cm			Well-trained 120.8 ± 8.7 cm *										
	Maulder, Bradshaw [40]	National and regional level sprinters 1.04 ± 0.03 m													
	Mero, Kuitunen [19]	Block angle 40° 1.09 ± 0.06 m			Block angle 65° 1.06 ± 0.06 m										
	Čoh, Jost [13]	Slovene national sprinters 100.85 ± 9.79 cm						Slovene national sprinters 98.64 ± 6.74 cm							
First step contact time (ms) (s)	Werkhausen, Willwacher [43]							Germany national sprinters 0.20 ± 0.02 s							
	Graham-Smith, Colyer [39]	Seniors 0.195 ± 0.022 s			Juniors 0.202 ± 0.024 s										
	Sandamas, Gutierrez-Farewik [24]									Skating condition 0.21 ± 0.01 s			Narrow condition 0.20 ± 0.01 s		
	Aeles, Jonkers [9]									Adult sprinters 0.191 ± 0.024 s			Young sprinters 0.199 ± 0.023 s		
	Ciacci, Merni [38]	World-class 0.210 ± 0.035 s			Elite 0.176 ± 0.008 s			World-class 0.225 ± 0.034 s	Elite 0.166 ± 0.017 s						
		Independent of category 0.189 ± 0.027 s						Independent of category 0.190 ± 0.038 s							
	Coh, Peharec [5]	Faster sprinters 170 ± 18.17 ms			Slower sprinters 174 ± 16.94 ms										
	Aerenhouts, Delecluse [1]	Elite Seniors 173 ± 67 ms			Elite Juniors 199 ± 24 ms			Elite Seniors 196 ± 62 ms	Elite Juniors 210 ± 17 ms						
	Slawinski, Bonnefoy [7]	Elite 0.173 ± 0.010 s			Well-trained 0.167 ± 0.011 s										
	Maulder, Bradshaw [40]	National and regional level sprinters 0.20 ± 0.02 s													
	Mero, Kuitunen [19]	Block angle 40° 0.185 ± 0.020 s			Block angle 65° 0.197 ± 0.019 s										
First flight time (ms) (s)	Ciacci, Merni [38]	World-class 0.045 ± 0.025 s			Elite 0.064 ± 0.009 s			World-class 0.045 ± 0.025 s	Elite 0.085 ± 0.011 s						
		Independent of category 0.056 ± 0.019 s						Independent of category 0.069 ± 0.027 s							
	Bezodis, Salo [3]	World-class to university sprinters 0.073 ± 0.022 s													
	Rabita, Dorel [22]	Elite 81 ± 13 ms			Sub-elite 70 ± 25 ms										
	Slawinski, Bonnefoy [7]	Elite 0.093 ± 0.009 s			Well-trained 0.087 ± 0.021 s										
	Maulder, Bradshaw [40]	National and regional level sprinters 0.07 ± 0.01 s													
Horizontal CM position—first step touchdown (cm) ^(a)^	Slawinski, Bonnefoy [7]	Elite 68.5 ± 4.7			Well-trained 58.0 ± 8.1 *										
Normalized first step touchdown distance ^(b)^	Bezodis, Salo [3]	World-class to university sprinters −0.20 ± 0.07													
Horizontal CM position—first step takeoff (cm) ^(a)^	Slawinski, Bonnefoy [7]	Elite 137.1 ± 9.0			Well-trained 120.8 ± 8.7 *										
First step resultant velocity (m·s^−1^)	Debaere, Vanwanseele [15]									Adult sprinters 4.34 ± 0.25		U18 sprinters 4.06 ± 0.24 ^#^		U16 sprinters 4.01 ± 0.25 ^#^	
	Slawinski, Dumas [8]									Bunched start 3.81 ± 0.18		Medium start 3.85 ± 0.16		Elongated start 3.90 ± 0.15	
	Slawinski, Bonnefoy [7]	Elite 4.69 ± 0.15			Well-trained 4.42 ± 0.11 *										
	Čoh, Jost [13]	Slovene national sprinters 4.48 ± 0.29						Slovene national sprinters 4.29 ± 0.18							
	Mero [18]	Trained sprinters 4.65 ± 0.28													
First step horizontal velocity (touchdown) (m·s^−1^)	Sandamas, Gutierrez-Farewik [24]									Skating condition 3.10 ± 0.16			Narrow condition 3.08 ± 0.16		
First step horizontal velocity (takeoff) (m·s^−1^)	Graham-Smith, Colyer [39]	Seniors 4.60 ± 0.23			Juniors 4.39 ± 0.21										
	Sandamas, Gutierrez-Farewik [24]									Skating condition 4.37 ± 0.18			Narrow condition 4.32 ± 0.15 *		
	Debaere, Delecluse [6]									Elite sprinters 4.28 ± 0.27					
	Čoh, Jost [13]	Slovene national sprinters 4.47 ± 0.29						Slovene national sprinters 4.25 ± 0.18 *							
First step change in horizontal velocity (m·s^−1^)	Werkhausen, Willwacher [43]							Germany national sprinters 1.09 ± 0.06							
	Aeles, Jonkers [9]									Adult sprinters 0.82 ± 0.39			Young sprinters 1.09 ± 0.25 *		
First step vertical velocity (m·s^−1^)	Graham-Smith, Colyer [39]	Seniors 0.46 ± 0.15			Juniors 0.54 ± 0.10										
	Chen, Wu [37]	Bunched start 0.27 ± 0.12		Medium start 0.28 ± 0.10		Elongated start 0.39 ± 0.13 *									
	Debaere, Delecluse [6]									Elite sprinters 0.67 ± 0.12					
	Slawinski, Bonnefoy [7]	Elite 0.35 ± 0.03			Well-trained 0.42 ± 0.09										
	Čoh, Jost [13]	Slovene national sprinters 0.37 ± 0.19 *						Slovene national sprinters 0.52 ± 0.10 *							
First step CM projection angle (°) ^(c)^	Graham-Smith, Colyer [39]	Seniors 5.7 ± 1.9			Juniors 7.1 ± 1.4 ^§§^										
First step takeoff angle (°) ^(d)^	Maulder, Bradshaw [40]	National and regional level sprinters 43 ± 2													
Trunk angle at touchdown—first step (°) ^(e)^	Chen, Wu [37]	Bunched start 27.2 ± 5.4		Medium start 30.9 ± 4.1 *		Elongated start 29.9 ± 4.7									
	Maulder, Bradshaw [40]	National and regional level sprinters 32 ± 8													
Hip angle at touchdown—first step (°)	Bezodis, Salo [3]	World-class to university sprinters 95 ± 9 ^(f)^													
	Debaere, Delecluse [6]									Elite sprinters 121.2 ± 11.3 ^(g)^					
Knee angle at touchdown—first step (°)	Bezodis, Salo [3]	World-class to university sprinters 101 ± 7 ^(h)^													
	Debaere, Delecluse [6]									Elite sprinters 111.6 ± 9.1 ^(h)^					
Ankle angle at touchdown—first step (°)	Bezodis, Salo [3]	World-class to university sprinters 96 ± 7 ^(i)^													
	Debaere, Delecluse [6]									Elite sprinters 70.6 ± 5.8 ^(i)^					
Maximal plantar-flexion—first step (°)										Elite sprinters 111.3 ± 11.2 ^(i)^					
Knee angle at takeoff—first step (°)										Elite sprinters 165.2 ± 20.6 ^(h)^					
Hip angle at takeoff—first step (°)										Elite sprinters 180.6 ± 20.9 ^(g)^					
Trunk angle at takeoff—first step (°) ^(c)^	Chen, Wu [37]	Bunched start 30.5 ± 6.9		Medium start 31.9 ± 6.2		Elongated start 32.5 ± 6.0									
	Maulder, Bradshaw [40]	National and regional level sprinters 32 ± 8													
Hip ROM extension—first step (°)	Aeles, Jonkers [9]									Adult sprinters 64.50 ± 13.08			Young sprinters 69.45 ± 9.53		
Knee ROM extension—first step (°)										Adult sprinters 60.09 ± 7.24			Young sprinters 58.24 ± 6.10		
Ankle ROM dorsiflexion—first step (°)	Werkhausen, Willwacher [43]							Germany national sprinters 17 ± 3							
Ankle ROM plantar flexion—first step (°)								Germany national sprinters 45 ± 6							
	Aeles, Jonkers [9]									Adult sprinters 59.05 ± 7.40			Young sprinters 50.96 ± 9.39 *		
Peak foot linear velocity (from the start to the first step) (m·s^−1^)	Chen, Wu [37]	Bunched start 6.31 ± 0.48		Medium start 6.66 ± 0.55 *		Elongated start 6.79 ± 0.99									
**Second Step**															
Second step length (m) (cm)	Cavedon, Sandri [12]									Usual condition 1.15 ± 0.14 nor. to leg length			Anthropometric condition 1.19 ± 0.12 nor. to leg length		
	Ciacci, Merni [38]	World-class 1.143 ± 0.105 m			Elite 1.057 ± 0.150 m			World-class 1.098 ± 0.104 m	Elite 1.078 ± 0.181 m						
		Independent of category 1.091 ± 0.135 m						Independent of category 1.086 ± 0.148 m							
	Coh, Peharec [5]	Faster sprinters 1.03 ± 0.12 m			Slower sprinters 0.98 ± 0.33 m										
	Debaere, Vanwanseele [15]									Adult sprinters 1.09 ± 0.06 m		U18 sprinters 1.01 ± 0.08 m ^#^		U16 sprinters 1.02 ± 0.08 m ^#^	
	Chen, Wu [37]	Bunched start 2.02 ± 0.18 m		Medium start 2.08 ± 0.18 m		Elongated start 2.10 ± 0.19 m									
	Milanese, Bertucco [41]									Rear knee angle @ 90° 1.96 ± 0.17 m		@ 115° 1.94 ± 0.12 m		@ 135° 1.93 ± 0.17 m	
	Aerenhouts, Delecluse [1]	Elite Seniors 148 ± 25 cm			Elite Juniors 130 ± 20 cm			Elite Seniors 130 ± 14 cm	Elite Juniors 127 ± 11 cm						
	Slawinski, Bonnefoy [7]	Elite 106.6 ± 5.9 cm			Well-trained 105.3 ± 6.3 cm										
	Maulder, Bradshaw [40]	National and regional level sprinters 1.08 ± 0.13 m													
	Čoh, Jost [13]	Slovene national sprinters 1.30 ± 0.51 m						Slovene national sprinters 1.06 ± 0.60 m							
Second step contact time (ms) (s)	Werkhausen, Willwacher [43]							Germany national sprinters 0.17 ±0.02 s							
	Graham-Smith, Colyer [39]	Seniors 0.173 ± 0.018 s			Juniors 0.173 ± 0.020 s										
	Ciacci, Merni [38]	World-class 0.170 ± 0.026 s			Elite 0.148 ± 0.008 s			World-class 0.180 ± 0.016 s	Elite 0.148 ± 0.013 s						
		Independent of category 0.157 ± 0.020 s						Independent of category 0.161 ± 0.021 s							
	Coh, Peharec [5]	Faster sprinters 157 ± 15.42 ms			Slower sprinters 149 ± 18.87 ms										
	Aerenhouts, Delecluse [1]	Elite Seniors 173 ± 28 ms			Elite Juniors 169 ± 20 ms			Elite Seniors 173 ± 19 ms	Elite Juniors 283 ± 23 ms						
	Slawinski, Bonnefoy [7]	Elite 0.138 ± 0.031 s			Well-trained 0.145 ± 0.016 s										
	Maulder, Bradshaw [40]	National and regional level sprinters 0.18 ± 0.03 s													
Horizontal CM position—second step touchdown (cm) ^(a)^	Slawinski, Bonnefoy [7]	Elite 168.2 ± 11.3			Well-trained 156.9 ± 12.4										
Horizontal CM position—second step takeoff (cm) ^(a)^		Elite 243.6 ± 13.9			Well-trained 224.9 ± 12.0 *										
Second step velocity (m·s^−1^)		Elite 5.50 ± 0.26			Well-trained 5.25 ± 0.13 *										
	Čoh, Jost [13]	Slovene national sprinters 5.40 ± 0.24						Slovene national sprinters 5.01 ± 0.29 **							
Second step horizontal velocity (m·s^−1^)	Graham-Smith, Colyer [39]	Seniors 5.48 ± 0.26			Juniors 5.27 ± 0.26										
	Debaere, Delecluse [6]									Elite sprinters 5.19 ± 0.30					
	Čoh, Jost [13]	Slovene national sprinters 5.38 ± 0.24						Slovene national sprinters 4.99 ± 0.29 **							
Second step change in horizontal velocity (m·s^−1^)	Werkhausen, Willwacher [43]							Germany national sprinters 1.12 ± 0.07							
Second step vertical velocity (m·s^−1^)	Graham-Smith, Colyer [39]	Seniors 0.54 ± 0.10			Juniors 0.62 ± 0.12										
	Debaere, Delecluse [6]									Elite sprinters 0.70 ± 0.17					
	Slawinski, Bonnefoy [7]	Elite 0.35 ± 0.05			Well-trained 0.45 ± 0.07 *										
	Čoh, Jost [13]	Slovene national sprinters 0.45 ± 0.18						Slovene national sprinters 0.50 ± 0.10							
Second step CM projection angle (°) ^(a)^	Graham-Smith, Colyer [39]	Seniors 4.9 ± 1.3			Juniors 6.8 ± 1.4 ^§§^										
Second step take off angle (°) ^(b)^	Maulder, Bradshaw [40]	National and regional level sprinters 46 ± 2													
Ankle angle at touch-down—second step (°)	Debaere, Delecluse [6]									Elite sprinters 72.4 ± 7.1 ^(i)^					
Ankle ROM dorsiflexion—second step (°)	Werkhausen, Willwacher [43]							Germany national sprinters 18 ± 3							
Ankle ROM plantarflexion—second step (°)								Germany national sprinters 44 ± 5							
Maximal plantarflexion—second step (°)	Debaere, Delecluse [6]									Elite sprinters 107.1 ± 15.0 ^(i)^					
Knee angle at touch-down—second step (°)										Elite sprinters 115.6 ± 6.2 ^(h)^					
Knee angle at takeoff—second step (°)										Elite sprinters 163.6 ± 17.7 ^(h)^					
Hip angle at touch-down—second step (°)										Elite sprinters 124.48 ± 11.3 ^(g)^					
Hip angle at takeoff—second step (°)										Elite sprinters 181.1 + 20.0 ^(g)^					
Trunk angle at touch-down—second step (°) ^(c)^	Chen, Wu [37]	Bunched start 33.4 ± 7.0		Medium start 34.9 ± 5.9		Elongated start 36.7 ± 5.9 *									
	Maulder, Bradshaw [40]	National and regional level sprinters 44 ± 8													
Peak foot linear velocity (from start to second step) (m·s^−1^)	Chen, Wu [37]	Bunched start 8.51 ± 0.58		Medium start 8.72 ± 0.40		Elongated start 8.68 ± 0.61 *									
**First and Second Steps**															
Step frequency First to second step (Hz)	Maulder, Bradshaw [40]	National and regional level sprinters 4.2 ± 0.3													
Minimal step frequency (Hz) ^(j)^	Rabita, Dorel [22]	Elite 3.94 ± 0.44			Sub-elite 3.90 ± 0.44										
Maximal step frequency (Hz) ^(j)^		Elite 4.95 ± 0.12			Sub-elite 4.80 ± 0.30 ^§§^										
Maximal CM horizontal acceleration (m·s^−2^)	Debaere, Delecluse [14]									First stance 0.36 ± 0.05			Second stance 0.23 ± 0.04 ***		
Maximal CM vertical acceleration (m·s^−2^)										First stance 0.28 ± 0.08			Second stance 0.25 ± 0.05 *		
Net induced acceleration (m·s^−2^)										First stance 501.4 ± 164.4 (33.2% horizontal/66.8% vertical)			Second stance 367.7 ± 36.7 *** (36.3% horizontal/63.7% vertical)		

CM—center of mass; ^(a)^ horizontal distance relative to stat line: ^(b)^ represents the horizontal distance (divided by leg length) between the CM and the stance leg metatarsal-phalangeal joint (negative value means that foot is behind the CM; ^(c)^ center of mass projection angle is calculated as the resultant direction from the horizontal and vertical block exit velocities of the center of mass; ^(d)^ the angle, measured relative to the horizontal, between the line passing through the most front part of the contact foot and the CG during takeoff; ^(e)^ the angle, measured relative to the horizontal, between the line passing through the hip and shoulder (trunk segment) of the support leg; ^(f)^ internal angle between the thigh and trunk in flexion/extension plane; ^(g)^ relative angle between the pelvis and the thigh according the Biomechanical Convention [53]; ^(h)^ relative angle between the thigh and the shank according the Medical Convention [53]; ^(i)^ relative angle between the shank and the foot according the Biomechanical Convention [53]; ^(j)^ data referring to the values recorded in the entire acceleration phase (0–40 m) excluding the block phase.

**Table A5:** Summary of the kinetic variables in the “first two steps”. Data are the magnitude of the mean ± SD presented in the reviewed studies. Groups are male and mixed (when authors joined data without discriminating by sex) sprinters. Studies are listed, in each variable, in reverse chronological order, followed by alphabetically for studies published in the same year. Data, terms, conditions, and sprinters’ performance levels are presented according to the original authors. Statistical differences between groups are marked with asterisks (* *p* < 0.05; *** *p* < 0.001; ^#^ significant different from adults; ^ƥ^ different from descending phase—*p* < 0.05; ^§^ small effect size [0.2–0.6] of 90% confidence intervals; ^Ϯ^ significantly greater compared with either leg in the block phase).

First and Second Steps Kinetics			Male		Mixed					
**First Step**										
GRFs	Relative resultant GRF (N·kg^–1^)	Sandamas, Gutierrez-Farewik [24]			Skating condition 1.51 ± 0.10 BW ^(a)^			Narrow condition 1.49 ± 0.12 BW ^(a)^		
		Otsuka, Shim [42]	Well-trained sprinters 14.93 ± 0.79	Trained sprinters 14.62 ± 1.44						
	Maximal horizontal force (N)	Aeles, Jonkers [9]			Adult sprinters 488.47 ± 268.16			Young sprinters 552.91 ± 147.40		
	Average horizontal force (N)				Adult sprinters 289.63 ± 163.32			Young sprinters 333.15 ± 94.26		
	Relative maximal horizontal force (N·kg^−1^)				Adult sprinters 7.09 ± 3.28			Young sprinters 9.02 ± 2.00 *		
	Relative average horizontal force (N·kg^−1^)	Sandamas, Gutierrez-Farewik [24]			Skating condition 0.64 ± 0.06 BW ^(a)^			Narrow condition 0.63 ± 0.04 BW ^(a)^		
		Aeles, Jonkers [9]			Adult sprinters 4.21 ± 2.04			Young sprinters 5.43 ± 1.28 *		
		Otsuka, Shim [42]	Well-trained sprinters 5.87 ± 0.35	Trained sprinters 5.48 ± 0.77						
	Maximal ratio of hori-zontal to total GRF (%)	Aeles, Jonkers [9]			Adult sprinters 61.31 ± 20.75			Young sprinters 78.00 ± 12.37 *		
	Mean ratio of horizontal force to total GRF (%)				Adult sprinters 28.49 ± 12.16			Young sprinters 37.04 ± 6.37 *		
	Relative average vertical force (N·kg^−1^)	Otsuka, Shim [42]	Well-trained sprinters 13.59 ± 0.82	Trained sprinters 13.43 ± 1.35						
Power	Relative average hori-zontal external power—first step (W·kg^−1^)	Graham-Smith, Colyer [39]	Seniors 25.1 ± 3.6	Juniors 23.1 ± 6 ^§^						
Impulses	Absolute Impulse (N·s)	Slawinski, Bonnefoy [7]	Elite 104.8 ± 16.5	Well-trained 78.6 ± 6.3 *						
	Net normalized horizontal impulse (m·s^−1^)	Sandamas, Gutierrez-Farewik [24]			Skating condition 1.29 ± 0.06			Narrow condition 1.26 ± 0.04		
	Normalized horizontal braking impulse (m·s^−1^)				Skating condition 0.04 ± 0.04			Narrow condition 0.03 ± 0.02		
	Normalized horizontal propulsive impulse (m·s^−1^)				Skating condition 1.33 ± 0.06			Narrow condition 1.29 ± 0.05 *		
	Normalized vertical impulse (m·s^−1^)				Skating condition 0.71 ± 0.18			Narrow condition 0.71 ± 0.28		
	Normalized mediolateral impulse (m·s^−1^)				Skating condition 0.33 ± 0.10			Narrow condition 0.17 ± 0.10 *		
Joint moments	Relative ankle joint moment (Plantar Flexion) (N·m·kg^−1^)	Aeles, Jonkers [9]			Adult sprinters 0.19 ± 0.05			Young sprinters 0.22 ± 0.07		
		Debaere, Vanwanseele [15]			Adult sprinters 0.19 ± 0.07		U18 sprinters 0.24 ± 0.06		U16 sprinters 0.21 ± 0.12	
		Debaere, Delecluse [6]			Elite sprinters 0.20 ± 0.03 N·m·N^−1^					
	Relative knee joint moment (extension) (N·m·kg^−1^)	Aeles, Jonkers [9]			Adult sprinters 0.29 ± 0.10			Young sprinters 0.21 ± 0.09 *		
		Debaere, Vanwanseele [15]			Adult sprinters 0.30 ± 0.11		U18 sprinters 0.18 ± 0.08 ^#^		U16 sprinters 0.18 ± 0.09 ^#^	
		Debaere, Delecluse [6]			Elite sprinters 0.20 ± 0.04 N·m·N^−1^					
	Relative hip joint moment (extension) (N·m·kg^−1^)	Aeles, Jonkers [9]			Adult sprinters 0.24 ± 0.08			Young sprinters 0.22 ± 0.07		
		Debaere, Vanwanseele [15]			Adult sprinters 0.50 ± 0.22		U18 sprinters 0.34 ± 0.10 ^#^		U16 sprinters 0.42 ± 0.09	
		Debaere, Delecluse [6]			Elite sprinters 0.33 ± 0.15 N·m·N^−1^					
	Relative hip joint moment (flexion) (N·m·kg^−1^)	Aeles, Jonkers [9]			Adult sprinters 0.41 ± 0.22			Young sprinters 0.26 ± 0.12 *		
		Debaere, Delecluse [6]			Elite sprinters 0.42 ± 0.16 N·m·N^−1^					
	Normalized peak ankle joint moment (extension)	Brazil, Exell [4]	Athletic sprinters 0.388 ± 0.035 ^(b)^ ^Ϯ^							
	Normalized peak knee joint moment (extension)		Athletic sprinters 0.242 ± 0.068 ^(b)^							
	Normalized peak hip joint moment (extension)		Athletic sprinters 0.330 ± 0.071 ^(b)^							
	Joint moments contributi-on to body propulsion (%)	Debaere, Delecluse [14]			Hip joint 10.3		Knee joint 9.6		Ankle joint 67.1 ***	
	Joint moments contribu—tion to body lift (%)				Hip joint 12.3		Knee joint 38.1		Ankle joint 49.6	
	Ankle joint stiffness as—cending phase (N·m/°) ^(c)^	Aeles, Jonkers [9]			Adult sprinters 6.64 ± 2.01			Young sprinters 7.35 ± 3.12 ^ƥ^		
	Ankle joint stiffness des— cending phase (N·m/°) ^(d)^				Adult sprinters 2.27 ± 0.62			Young sprinters 2.85 ± 1.23 ^ƥ^		
Joint powers	Relative ankle peak power (W·N^−1^)	Brazil, Exell [4]	Athletic sprinters 1.093 ± 0.069 ^(b)^ ^Ϯ^							
		Debaere, Vanwanseele [15]			Adult sprinters 1.79 ± 0.96		U18 sprinters 2.30 ± 1.02		U16 sprinters 2.19 ± 1.46	
	Relative knee peak power (W·N^−1^)	Brazil, Exell [4]	Athletic sprinters 0.468 ± 0.145 ^(b)^							
		Debaere, Vanwanseele [15]			Adult sprinters 3.03 ± 1.24		U18 sprinters 1.31 ± 0.66 ^#^		U16 sprinters 1.12 ± 1.2 ^#^	
	Relative hip peak power (W·N^−1^)	Brazil, Exell [4]	Athletic sprinters 0.908 ± 0.185 ^(b)^ ^Ϯ^							
		Debaere, Vanwanseele [15]			Adult sprinters 3.79 ± 0.95		U18 sprinters 4.56 ± 1.42		U16 sprinters 4.33 ± 0.96	
	Relative average hori-zontal external power (W·kg^−1^)	Graham-Smith, Colyer [39]	Seniors 25.1 ± 3.6	Juniors 23.1 ± 6 ^§^						
	Joint moments contributi-on to body propulsion (%)	Debaere, Delecluse [14]			Hip joint 10.3		Knee joint 9.6		Ankle joint 67.1 ***	
	Joint moments contributi-on to body lift (%)				Hip joint 12.3		Knee joint 38.1		Ankle joint 49.6 ***	
Joint work	Ankle negative work (J·kg^−1^)	Werkhausen, Willwacher [43]			Germany national sprinters −0.32 ± 0.14 ^(e)^					
	Ankle positive work (J·kg^−1^)				Germany national sprinters 1.58 ± 0.17 ^(e)^					
**Second Step**										
GRFs	Relative resultant GRF (N·kg^−1^)	Otsuka, Shim [42]	Well-trained sprinters 14.93 ± 0.79	Trained sprinters 14.62 ± 1.44						
	Relative average horizontal force (N·kg^−1^)		Well-trained sprinters 4.83 ± 0.70	Trained sprinters 4.79 ± 0.47						
	Relative average vertical force (N·kg^−1^)		Well-trained sprinters 13.22 ± 1.15	Trained sprinters 13.72 ± 1.18						
Power	Relative average horizontal external power—second step (W·kg^−1^)	Graham-Smith, Colyer [39]	Seniors 26.7 ± 3.6	Juniors 24.9 ± 4.5 ^§^						
Impulses	Absolute impulse (N·s)	Slawinski, Bonnefoy [7]	Elite 75.0 ± 15.8	Well-trained 55.9 ± 9.4 *						
Joint moments	Relative ankle joint moment (plantar flexion) (N·m·N^−1^)	Debaere, Delecluse [6]			Elite sprinters 0.23 ± 0.05					
	Relative knee joint moment (extension)(N·m·N^−1^)				Elite sprinters 0.10 ± 0.04					
	Relative hip joint moment (extension) (N·m·N^−1^)				Elite sprinters 0.43 ± 0.01					
	Relative hip joint moment (flexion) (N·m·N^−1^)				Elite sprinters 0.20 ± 0.06					
	Joint moments contributi-on to body propulsion (%)	Debaere, Delecluse [14]			Hip joint 0		Knee joint 7.1		Ankle joint 92.9 ***	
	Joint moments contributi-on to body lift (%)				Hip joint 0		Knee joint 23.8		Ankle joint 72.6 ***	

CM—center of mass; GRF—ground reaction forces; ^(a)^ normalized to body weight; ^(b)^ joint data normalized to the mass and the leg length of the sprinter; ^(c)^ ankle joint stiffness was calculated during the increase in ankle joint moment; ^(d)^ ankle joint stiffness was calculated during the decrease in ankle joint moment; ^(e)^ only elite female data.
